# Deciphering the molecular mechanism of the cancer formation by chromosome structural dynamics

**DOI:** 10.1371/journal.pcbi.1009596

**Published:** 2021-11-09

**Authors:** Xiakun Chu, Jin Wang

**Affiliations:** 1 Department of Chemistry, State University of New York at Stony Brook, Stony Brook, New York, United States of America; 2 Department of Physics and Astronomy, State University of New York at Stony Brook, Stony Brook, New York, United States of America; Weizmann Institute of Science, ISRAEL

## Abstract

Cancer reflects the dysregulation of the underlying gene network, which is strongly related to the 3D genome organization. Numerous efforts have been spent on experimental characterizations of the structural alterations in cancer genomes. However, there is still a lack of genomic structural-level understanding of the temporal dynamics for cancer initiation and progression. Here, we use a landscape-switching model to investigate the chromosome structural transition during the cancerization and reversion processes. We find that the chromosome undergoes a non-monotonic structural shape-changing pathway with initial expansion followed by compaction during both of these processes. Furthermore, our analysis reveals that the chromosome with a more expanding structure than those at both the normal and cancer cell during cancerization exhibits a sparse contact pattern, which shows significant structural similarity to the one at the embryonic stem cell in many aspects, including the trend of contact probability declining with the genomic distance, the global structural shape geometry and the spatial distribution of loci on the chromosome. In light of the intimate structure-function relationship at the chromosomal level, we further describe the cell state transition processes by the chromosome structural changes, suggesting an elevated cell stemness during the formation of the cancer cells. We show that cell cancerization and reversion are highly irreversible processes in terms of the chromosome structural transition pathways, spatial repositioning of chromosomal loci and hysteresis loop of contact evolution analysis. Our model draws a molecular-scale picture of cell cancerization from the chromosome structural perspective. The process contains initial reprogramming towards the stem cell followed by the differentiation towards the cancer cell, accompanied by an initial increase and subsequent decrease of the cell stemness.

## Author summary

Cancer is among the leading causes of human death. Cancer is regulated by the underlying regulatory network of gene expressions, which are in intimate relation to the 3D chromosome architectures. Numerous efforts have been spent on elucidating the chromosome structural variants in tumorigenesis, while the dynamical picture of how chromosomes structurally evolve during cancer formation is still missing. Here we integrate the Hi-C data into the polymer simulations to build the chromosome structural ensembles in the normal and cancer cells. Then we use a nonequilibrium landscape-switching model to simulate the chromosome structural dynamics during the cancerization and reversion processes. With quantified pathways, we show that the chromosomes at transient intermediate states in the cancer formation possess a significant degree of structural similarity to those at the stem cell. Our findings indicate the formation of stem-like states during cancer formation from the chromosome structural perspective. We draw a molecular-scale picture of the cancer formation, which contains initial reprogramming towards the stem cell followed by differentiation towards the cancer cell.

## Introduction

Currently, our knowledge of how cancer cells form and proliferate is still quite limited. In general, the development of cancer is controlled by the underlying gene regulatory network [1], which relies on the molecular interactions between the spatially organized genome and a broad class of proteins, including the transcription factors and chromatin remodelers [2]. As the structural scaffold for the genome function, the 3D genome architecture has been recognized to play an important role in regulating the gene expression [3–5], leading to the intimate structure-function relationships at the genomic level. Several studies have shown that alterations in chromosome structures and interactions can make significant contributions to the dysregulation of the gene expressions forming the specific cancer signatures [6–9]. These findings have fostered a view that the 3D context of the genome is the major player in the development and progression of cancer [10]. Therefore, investigating the disorganization of the 3D genome structure in cancer cells can provide the key to understanding the pathogenesis of cancer.

Although the chromosome structural variant, which is the major form of the genome instability, has been regarded as a hallmark of almost all human cancers [11–13], determining the genome structure has been a long-term challenge until the emergence of chromosome conformation capture (3C) techniques nearly two decades ago [14]. As an advanced derivative of the 3C, Hi-C measures the spatial proximity of the chromosomal loci across the entire genome in terms of the contact frequency map and often provides an ensemble description of the genome organization within a large number of cells [15, 16]. Recently, Hi-C techniques were applied to characterize the disorganization of the cancer genomes and determine their functional consequences [17]. Taberlay et al. observed the smaller-sized topologically associated domains (TADs) formed in prostate cancer cells than those in normal cells due to the emergence of additional domain boundaries. This further leads to alterations of TP53 tumor suppressor locus [18]. In addition, the effects of the structural disruptions within TADs on leading to various cancer types were established [19–21]. At the higher hierarchical level, Barutcu et al. found that the significant A/B compartment switching between the normal and breast cancer cell is associated with gene expression changes [22]. These studies have shown that the structural variants in the cancer genome occur throughout the genome sequence, and they have important impacts on the gene expression alterations for inducing cancer formation.

Increasing evidence indicates that cancer development is orchestrated by a small subpopulation of the cancer cells, namely the cancer stem (CS) cells. [23–26]. CS cells, which possess stem-like properties and functions, are capable of performing self-renewal, proliferation and differentiation. Thus they provide the driving forces for cancer progression [27, 28]. However, experimental identifications on the CS cell and the associated dynamics are non-trivial due to its rare population in the cancer cells [29]. The previous theoretical studies using gene regulatory work, which includes the cancer and developmental genetic markers as well as the interactions between them, provided a landscape view of how the cancer cell develops and CS cell forms [30, 31]. However, the work focused on simplified core gene regulatory networks to describe the cancer systems, so the results were restricted in the form of regulation pathways among several marker genes and this description is at the gene network level. Therefore, there is still a lack of a complete molecular chromosomal-structural level understanding of cancer development and CS cell formation. On the other hand, the strategies on inducing the cancer cell to normal cell by reversion [32] or transdifferentiation processes [33–35] are rapidly developing. These approaches have opened new avenues for cancer treatments, but the underlying mechanism of the cancer reversion is still unclear [36].

Despite the significant achievements made by the Hi-C technique on elucidating the spatial genome disorganization in the cancer cells [17], the temporal dynamic rearrangement of the genome structure during cancer development is still not available. The picture is fundamentally important as it may provide a molecular-level understanding of the cancer mechanism. Nevertheless, measuring the chromosome structural dynamics during the cancer cell developmental process is extremely challenging due to the spatial and temporal resolution limits of the current experiments. Owing to the rapid development of the 4D Nucleome program [37], in particular the time-course Hi-C technique, it has been possible to characterize the spatiotemporal organizations of the genome during different cellular processes [38–40]. Recently, the data probed at the discrete time-points along the biological processes in the experiments were used to model the structures of the chromosomes, and further implemented to construct the gradual and smooth trajectories through simulations [41, 42]. These data-driven approaches have provided useful information to interpret the experiments and moved further to characterize the relationship between the structure and function of the chromosome. However, the time-course Hi-C experiments are often laborious and expensive, and the measurements do not provide the real-time dynamics but in the form of ensemble-based data, which inevitably contain the cell state heterogeneity, thus impeding our understanding of the intermediate states during the transition [43, 44].

Here, we developed molecular dynamics models and performed associated simulations to investigate the dynamic cancer-related chromosome structural evolution, which provides the microscopic description of the cancer process. First, we applied the maximum entropy principle to incorporate the Hi-C data of the normal and cancer cells into two independent sets of simulations. These simulations generated two potentials for describing the chromosome structural ensembles that reproduced the Hi-C data, in the normal and cancer cells, respectively (S1 and S2 Figs) [45]. In the cell nucleus, the chromosome constantly experiences the nonequilibrium effects even at one cell state, leading to a nonequilibrium system. Theoretically, it has been demonstrated that the dynamics of the nonequilibrium system can be described using the concept of an effective landscape in some circumstances [46, 47]. Recent studies revealed that under an effective equilibrium landscape, the chromosome dynamics reproduces many aspects of kinetic behaviors as observed in experiments, including anomalous diffusion, viscoelasticity, and spatially coherent dynamics [48, 49]. Here, to examine whether the potentials accounting for chromosome structures obtained from maximum entropy principle simulations can also be used for describing the chromosome dynamics, we performed additional simulations under these two potentials and calculated the diffusion behaviors of the chromosome motions in the normal and cancer cells, respectively (S3 Fig). We observed sub-diffusivity of chromosome dynamics with the scaling exponents of the mean square displacement in good agreement with multiple experiments [50, 51]. Our results have suggested that these two potentials generated by the maximum entropy principle simulations for the chromosome in the normal and cancer cells can be further regarded as the effective energy landscapes that govern the chromosome conformational dynamics within the normal and cancer cells, respectively. Although each state can be described by an effective equilibrium landscape, the inter-basin dynamics of switching between the normal and cancer states often requires a significant amount of energy input [52] and therefore is a nonequilibrium process, which cannot be treated in an equilibrium way. This is the motivation and target of our current study. In this regard, the cancer-related chromosome structural transitions are further described by the connection between these two effective landscapes during the cell cancerization and reversion processes.

To bridge these two landscapes for describing transitions between the normal and cancer cells, we then used a landscape-switching model, which was developed to simulate the chromosome structural transition during the cell cycle [53] and the cell developmental processes [45]. Briefly, the chromosome structural dynamics at either normal or cancer cell was initially described by an effective equilibrium landscape generated by the data-driven maximum entropy principle simulation. Then, the cell state transition process was triggered by an instantaneous energy excitation that switches the system from one landscape to the other. This energy excitation implementation provides the extra energy for the inter-landscape switching, thus it breaks the detailed balance of the system, leading to the nonequilibrium process. Finally, the relaxation dynamics of the chromosome structure occur on the post-switching effective equilibrium landscape (see “Materials and methods”). The rationales for approximating the transitions between the normal and cancer cells to simple switches are based on the following two facts.

1. The cancer process often exhibits switch-like behavior between two steady cell states, in accordance with the landscape-switching model. The cell state transition system, including cancer, usually shows bistability at the initial and final cell states [54, 55]. The normal and cancer cells, deemed as the attractors on the cell developmental landscape, dictate the transition processes [31, 56, 57]. Increasing experimental evidence suggested that the cell state transitions likely undergo switching between the two stable cell states [58–61]. To accommodate these features, the model used a landscape-switching implementation to trigger cell cancerization and reversion. For practical correspondence, the switch mimics the roles of the genetic mutations and epigenetic modifications in initiating cancer processes [62, 63]. Although the individual genetic mutations and epigenetic modifications during the cancer processes may occur frequently, the changes of the cell phenotypes can only be steadily realized by the cooperative changes of the chromatin states at a global scale, leading to abrupt switches between the two distinct cell states [64, 65].

2. From the physical perspective, cell cancerization and reversion can be approximately described by the nonequilibrium nonadiabatic processes, quantified using the landscape-switching model. It has been recognized that the degree of the adiabaticity is determined by the interplay of the timescales for intra- and inter-landscape dynamics [66, 67]. A faster (slower) intra-landscape motion than inter-landscape hopping leads to a nonadiabatic (adiabatic) process. In reality, the normal and cancer cell states are stable and the transitions between them are impossible to occur spontaneously. In contrast, numerous internal and external factors associated with a significant amount of energy input work collaboratively to achieve the transition processes between these two cell states with distinct phenotypes [68]. These features lead to a significantly slower timescale for the cell waiting for the state transition (inter-landscape dynamics) than the cell relaxing within one stable state (intra-landscape dynamics). In analogous to the “surface hopping” method [69], we separated the simulations of the chromosome dynamics at one cell state and the nonequilibrium nonadiabatic inter-state switching dynamics, giving rise to the landscape-switching model.

We used the landscape-switching model to study the chromosome structural dynamics during the transitions between the human normal and cancer lung cells. We predicted that the chromosome can form more expanding structures than those at the normal and cancer cells during both of these processes but with different structural characteristics. Further analyses revealed that the chromosome at the transient intermediate state with a more expanding structure than those at the normal and cancer cells during the cancerization process share significant structural similarity to the one at the embryonic stem (ES) cell. This feature implies forming a cell state with the features of stemness or the CS cell during the cancerization process from the chromosome structural perspective. We observed the high irreversibility for cancerization and reversion through the quantified chromosome structural transition pathways, the spatial repositioning of chromosomal loci and the hysteresis loop of establishing the chromosomal contacts. These findings underlined distinct mechanisms for these two cancer cellular processes. The prediction of forming a stem-like intermediate cell state at the chromosomal-structural level during cancerization can be tested by the future time-course Hi-C experiments designed for the cancer developmental processes.

## Results

### Chromosome structural transitions during cancerization and reversion

We used the landscape-switching model to investigate the chromosome dynamics during the cell cancerization and reversion processes. The chromosome used in this study is the long arm of human chromosome 14 (20.5–106.1 Mb). We focused on the terminally differentiated human lung fibroblast cell (IMR90) and lung cancer cell (A549), and investigated the chromosome structural changes during the transitions between these two cell states. We note that there are no chromosome abnormalities, such as the chromosome deletion, duplication, inversion, substitution and translocation, occurring on the chromosome 14 in the A549 cell [17, 70]. This feature indicates that the structural differences of chromosome 14 between the IMR90 and A549 cells are all related to the intra-chromosome structural rearrangements and can be studied by molecular dynamics simulations.

**Fig 1:**
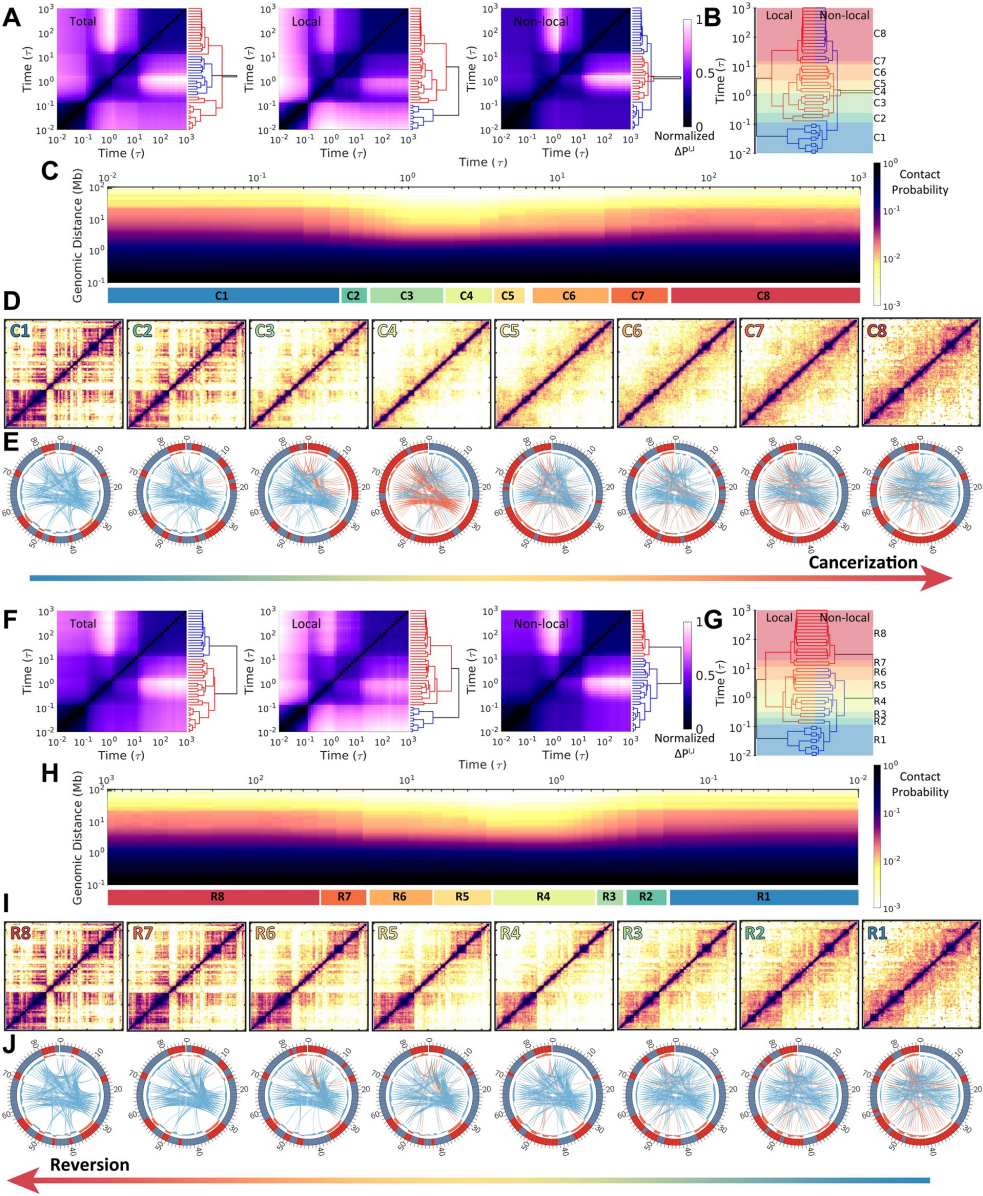
Chromosome structural transitions during cancerization of IMR90 (normal cell) to A549 (cancer cell) (*Upper*) and reversion of A549 to IMR90 (*Lower*). (A) Hierarchical clustering of the chromosomal contact probability among each time frame *t* = *I*, *J* during cancerization varied by total, local (< 2Mb), and non-local (≥ 2Mb) contact ranges. The clustering was performed on the contact map difference Δ*P*^*I*,*J*^ (see “**Materials and Models**”). (B) Reduced 8 stages (“C1”-“C8”) for the cancerization process based on the combination and comparison of the dendrograms of local and non-local Δ*P*^*I*,*J*^ established in (A). Thus the chromosomes within one stage possess relatively similar contact probability maps. (C) The change of contact probability *P*(*l*) versus genomic distance *l* in the chromosome during cancerization with the 8 stages indicated at the bottom. (D) Hi-C heat (contact probability) maps of the chromosome for the 8 stages during cancerization. (E) The circle plots of chromosomes for the 8 stages during cancerization [75]. The red and blue bands indicate the loci in compartments A and B, respectively. The compartment profiles are further shown as histograms near to the band plots. The connections show the long-range (> 5Mb) interactions which are identified by the *P*_*obs*_/*P*_*exp*_, where *P*_*obs*_ and *P*_*exp*_ are observed and expected contact probability, respectively [15]. The red, blue, and grey lines indicate the interactions between chromosomal loci within compartment A, within compartment B, and between compartment A and B. The line widths correspond to the logarithmic scale of the *P*_*obs*_/*P*_*exp*_ and only the top 200 weighted contacts are shown for better visualization. (F-J) are similar with (A-E) but for the reversion process of A549 to IMR90. Another reduced 8 stages during the reversion (“R1”-“R8”) are determined in (G).

The implementation of the landscape-switching model in molecular dynamics simulation is briefly summarized as follows. First, we iteratively fit a generic polymer model to reproduce the experimental Hi-C data through the maximum entropy principle simulation for the IMR90 and A549 cell, separately [71]. Previous studies have shown that the resulting potential not only captures the thermodynamics of the chromosome (i. e., Hi-C) [72–74], but also describes the correct kinetic properties of the chromosomal loci diffusion within one cell state (one phase in cell cycle or one cell state in cell differentiation/reprogramming) [48, 49]. Here, we termed the potential *V*(***r***|*S*) as the effective energy landscape for describing the chromosome dynamics in the normal or cancer cell, where ***r*** is the coordinate of the system at the cell state *S* (IMR90 or A549). Next, the simulation was set up with the chromosome exploring the structural dynamics under either the energy landscape of the normal (*V*(*r*|*IMR*90)) or cancer (*V*(***r***|*A*549)) cell. Then, the energy landscape underwent a switch from normal to cancer cell (*V*(***r***|*IMR*90) → *V*(***r***|*A*549)) or cancer to normal cell (*V*(***r***|*A*549) → *V*(***r***|*IMR*90)) to trigger the chromosome structural transition during cancerization or reversion, respectively. Finally, the chromosome dynamics was governed by the post-switching energy landscape of the cancer cell (*V*(***r***|*A*549)) or normal cell (*V*(***r***|*IMR*90)). The model allowed us to observe the chromosome transformation during the cancerization and reversion processes with affordable computational resources (Fig 1).

We performed hundreds of independent landscape-switching simulations starting from different chromosome structures in the normal (and cancer) cell state to investigate the chromosome structural transitions during the cancerization (and reversion) process (See “Materials and methods”). By comparing the differences of the contact probability formed through the pairwise chromosomal loci between time-series frames during the cancerization process (denoted as Δ*P*^*I*,*J*^, where *P* is contact probability and *I* and *J* are the time points), the transition can be reduced into two clusters according to the hierarchical clustering of Δ*P*^*I*,*J*^ along with the processing time (Fig 1A). However, the clustering dendrograms for the local and non-local chromosomal contacts are very different, indicating distinct behaviors of structural rearrangements at the local and non-local ranges. The separation of the two clusters at the local contacts occurs early at ∼ 0.1*τ*, where *τ* is the time unit in the simulation. In contrast, the non-local chromosomal contacts at the beginning of the transition are relatively similar to those at the late stages, and they are separated by the second cluster at ∼ 0.5–10*τ*. This is also reflected by the contact probability *P*(*l*) versus the genomic distance *l* between a pair of chromosomal loci (Fig 1C), where an apparent decrease of *P*(*l*) is observed at time ∼ 0.5–10*τ*.

By examining the clustering dendrogram patterns of the local and non-local contact formations in the chromosome, we further reduced the cancerization process into 8 stages (Fig 1B). As a result, chromosomes within one stage are structurally similar in terms of the local and non-local contacts. The contact maps of the 8 stages show a picture of how chromosomes perform the structural rearrangements in the caner formation process (Fig 1D). Interestingly, we found that as the cancerization proceeds, the chromosome heat maps show lower probabilities (more sparse contacts in heat maps) than those in both normal and cancer cells. The result implies that the chromosome structure is expanding during cancerization. Besides, we observed a complex manner of the chromosome in organizing the compartment change during cancerization shown by the colored bands in the circle plots (Fig 1E). From the stage “C1” to “C3”, the populations of the chromosomal loci in compartment A increase associated with an increasing quantity of contacts (lines in each circle plot) formed within compartment A. At the stage “C4”, the chromosomal loci are roughly assigned to compartment A and B by sequence and the long-range contacts are rare. This implies the extensive breaking of the contacts formed in the chromosome at the normal cell. From the stage “C5” to “C8”, the chromosomal compartment profiles and contact patterns gradually adapt to those at the cancer cell. Finally, we see that the chromosome in the cancer cell has more chromosomal loci in compartment A and forms more sparse contacts than the one in the normal cell. In the ES cells, chromosomes possess high populations of euchromatin, which maps with compartment A, to benefit the cell pluripotency and differentiation [76–78]. Our observation of the chromosome with populated expressive loci and open structure in the cancer cell resonates with the experimental evidence that the cancer cells possess certain contents of the “stemness” in favor of the self-renewal, differentiation, and proliferation [23, 79, 80].

Then we applied similar analyses into the reversion process. We found that the chromosome structural transition during the reversion process is not the simple reversal of the cancerization. From the chromosomal contact formation perspective, the reversion process can also be grouped into two clusters (Fig 1F). However, these two clusters are separated by the time in sequence from the dendrogram plots. In detail, the time for separating the two clusters at local contact (∼ 0.1*τ*) occurs much earlier than that at non-local contacts (∼ 10*τ*). This indicates that chromosome structurally evolves faster at local than non-local range during reversion. Interestingly, the time evolution of *P*(*l*) ∼ *l* also shows a chromosome structural expansion at the time ∼ 0.5–10*τ* (Fig 1H). However, the expansion appears to be less significant than that observed during cancerization. The chromosomal contact maps show distinct evolving ways deviated from the reversal of that in cancerization (Fig 1I). In particular, there is no significant compartment-switching during the reversion process, as the compartment undergoes gradual and moderate change, associated with reducing the contacts formed within compartment A (Fig 1J). Overall, we found that the chromosome structural transitions undergo initial expansions followed by compactions during both the cancerization and the reversion processes but through irreversible paths.

### Chromosome structural expansions during cancerization and reversion

**Fig 2:**
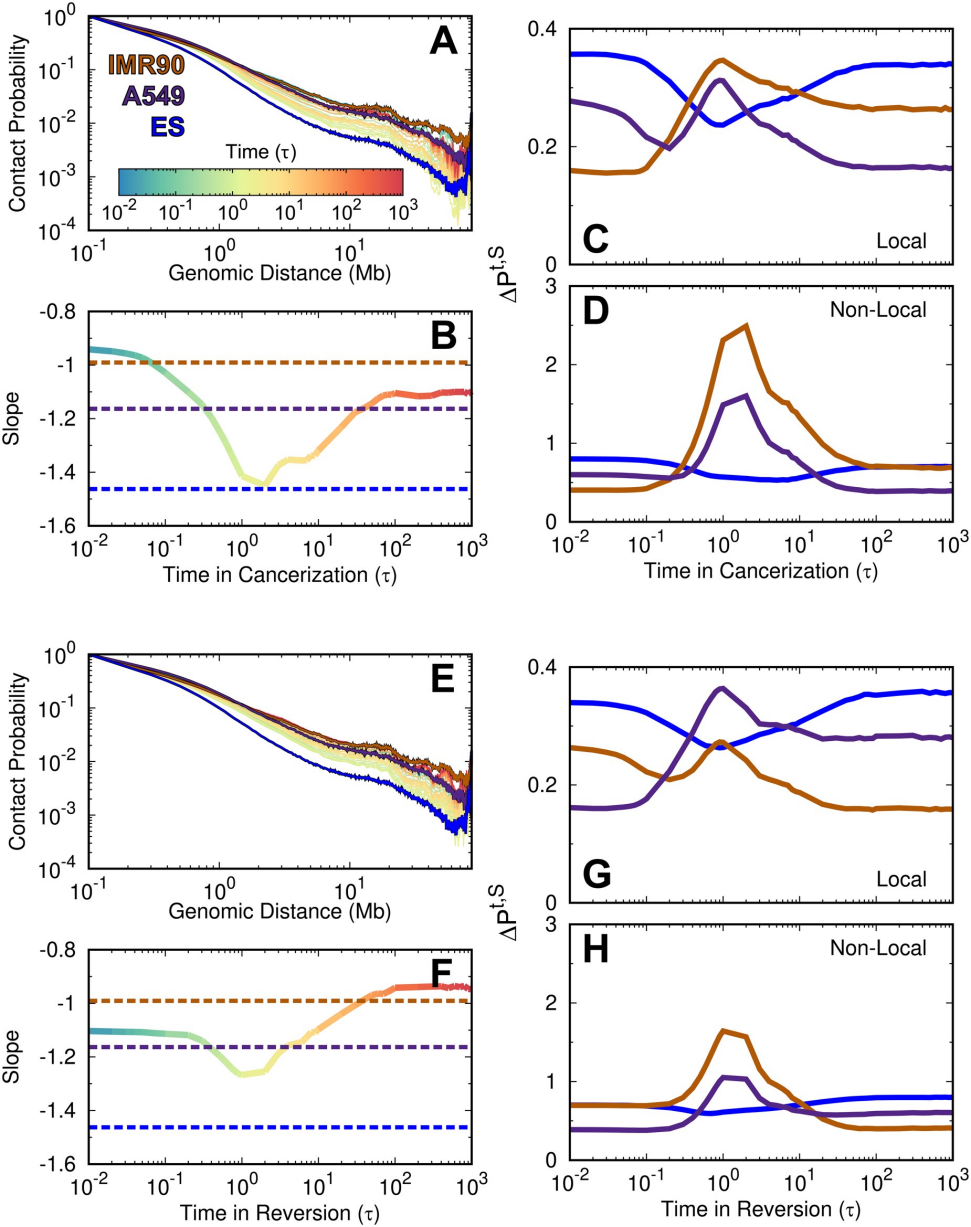
The time evolution of the chromosomal contact probability during cell cancerization and reversion. (A) The time evolution of contact probability *P*(*l*) versus genomic distance *l* during cancerization. The Hi-C data of the IMR90, A549, and ES cells are also plotted. (B) The time evolution of the slope in the logarithmic relation of *P*(*l*) ∼ *l* at the range of 0.5–7 Mb during cancerization. The differences of contact probability map during the cancerization process to the Hi-C data of IMR90, A549 and ES cells at the (C) local and (D) non-local ranges. (E-H) are the same with (A-D) but for the reversion process.

To examine the chromosome structural expansion during cancerization and reversion, we monitored the contact probability *P*(*l*) versus the genomic distance *l*. *P*(*l*) provides the information on how the chromosome organizes its structure along the genomic sequence distance, thus it dictates the polymer state of the chromosome [81, 82]. Chromosomes in both the normal and cancer cells show similar *P*(*l*) trends with higher curves, corresponding to more compacted structures, than the ones in the ES cell [78] (Fig 2A). This can also be inferred by the slope in the logarithmic relation of *P*(*l*) ∼ *l* (Fig 2B). As the normal cell converts to the cancer cell, the profile of *P*(*l*) (colored by time) initially goes down and approaches the one at the ES cell (blue line), then increases to the cancer cell (purple line). The result draws a picture of the chromosome expanding its structure followed by compaction during cancerization. To quantitatively measure this trend, we calculated the slope in the logarithmic relation of *P*(*l*) ∼ *l*. We see that the slope at ∼ 2*τ* has the lowest value and approximates to the value found at the ES cell. This implies that the chromosome with the maximum structural expansion possesses a similar contact formation scaling versus the genomic sequence distance with the one at the ES cell. In other words, the chromosome may form the chromosome structural characteristics of the ES cell during cancerization. Besides, it is worth noting that the chromosome in the cancer cell is more expanding than it is in the normal cell, as the *P*(*l*) plot for the cancer cell is slightly below the one for the normal cell.

To measure the similarity of the chromosomal contact formation with that in the ES cell during cancerization, we calculated the difference of the contact probability between the processing state at time point *t* during cancerization and the normal, cancer and ES cells at the local and non-local ranges (denoted as *P*^*t*,*S*^ in Fig 2C and 2D, see “Materials and methods”). We found that the chromosome at ∼ 0.5–10*τ*, when the chromosome appears to be more expanding than those at the normal and cancer cells, as indicated in Fig 2B, *P*^*t*,*S*^ of the normal and cancer increase significantly while *P*^*t*,*S*^ of the ES cell decreases. This implies that the contact formation in the chromosome with a more expanding structure than those at the normal and cancer cells, becomes similar to the one at the ES cell. The results indicate that the chromosome may go through stem-like structures to accomplish the cancerization process.

In contrast, the chromosome expansion in the reversion process from the cancer to normal cell is not significant (Fig 2E). When the chromosome has the most significant structural expansion with the lowest *P*(*l*), the profile of *P*(*l*) still visually deviates from the one in the ES cell. The steepest slope in *P*(*l*) ∼ *l* shows a slight decrease from that in the cancer cell and occurs at the time ∼ 0.5–3 *τ*. Besides, we can still observe that the similarity of the chromosomal contact formation between the processing state and the ES cell increases, but it is not as much as that observed during cancerization (Fig 2G and 2H). Therefore, the chromosome during the reversion process may possess less structural properties at the ES cell than during cancerization.

### Quantified chromosome structural transition pathways during cancerization and reversion

**Fig 3:**
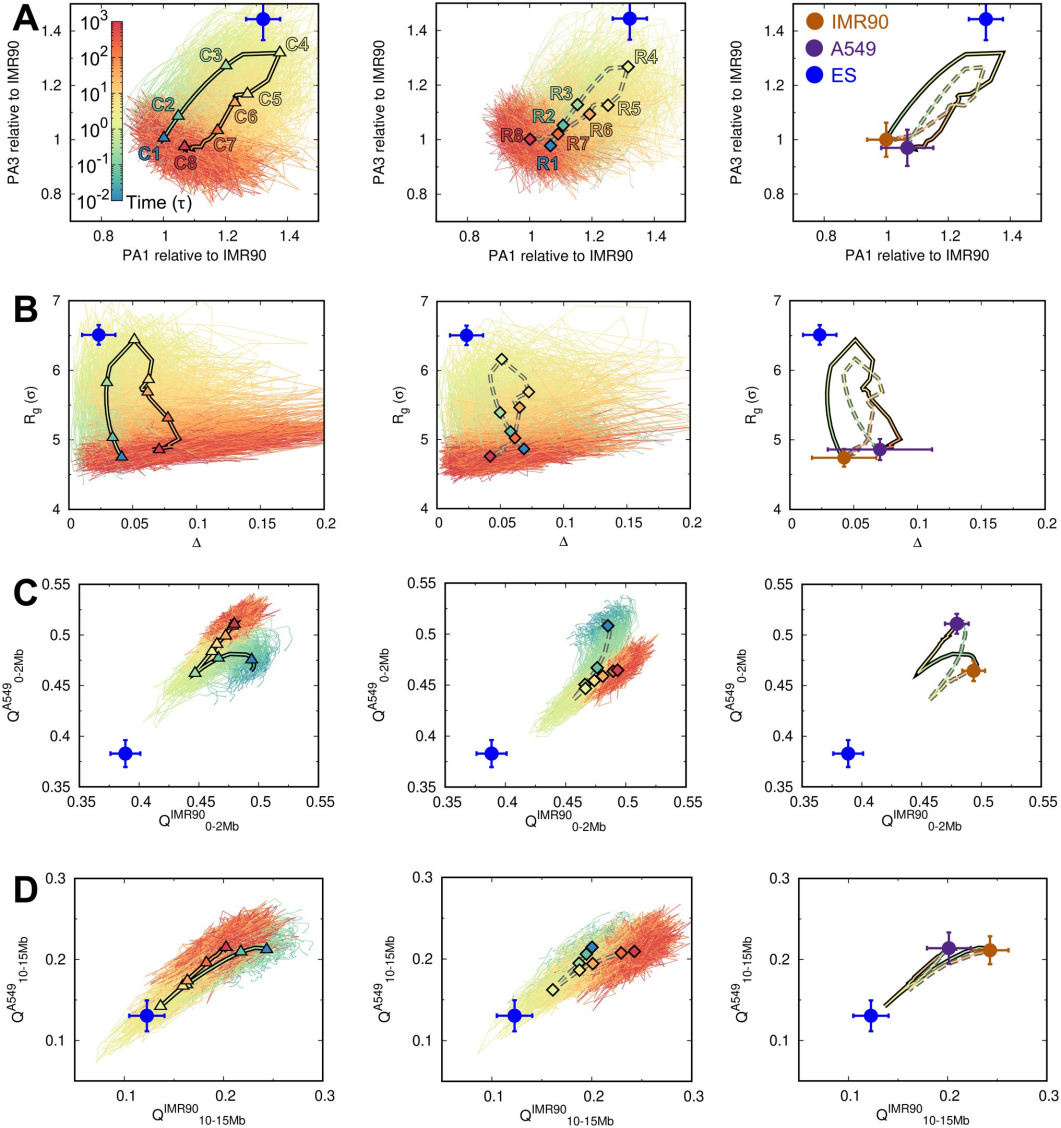
The pathways of chromosome structural transitions during cell cancerization and reversion. The pathways are projected onto the order parameters of the chromosome with all trajectories presented for cancerization (*Left*) and reversion (*Middle*). The 8 stages are indicated by triangles (“C1”-“C8” in cancerization, *Left*) and diamonds (“R1”-“R8” in reversion, *Middle*) on the averaged pathways of the cancerization (solid line) and reversion (dashed line), respectively. The averaged pathways are additionally shown and colored by time (*Right*). The quantities of the chromosome in the IMR90, A549, and ES cells are correspondingly placed as brown, purple and blue points, respectively. The pathways are projected onto (A) the extensions of the longest and the shortest principal axes (PA1 and PA3), (B) the radius of gyration (*R*_*g*_) and the aspheric quantity (Δ), contact similarity in terms of the fraction of native contact *Q* to the IMR90 and A549 at (C) local range (0–2Mb), and (D) long-range (10–15Mb). The data of the ES cell are obtained from our previous work [45].

In order to obtain a quantitative picture of how the chromosome structural transitions occur during the cancerization and reversion processes, we projected all the transition trajectories as well as the averages onto several order parameters, which describe the shape of the chromosome structure and contact formation at various ranges (Fig 3 and S4 and S5 Figs). We first used the extension lengths of the chromosome structure along the longest and shortest principal axes (PA1 and PA3) [73] (Fig 3A). We found that the chromosomes at the normal and cancer cells show very similar extension lengths. However, changing the shapes of the chromosomes does not follow the direct straight line connecting the initial and final states during both the cancerization and reversion processes. In contrast, there is an increase followed by the decrease of shape extension on the chromosome structure along with both PAs during both the cancerization and reversion processes. In addition, the individual pathways show high stochasticity, reminiscent of the stochastic dynamics in cell development [83]. From the averaged pathways, we see that the transitions of these two processes do not follow the same route. This is also observed by projecting the pathways onto the radius of gyration (*R*_*g*_) and aspheric quantity (Δ). Δ measures the asphericity of the chromosome structure, and the deviation of Δ from 0 measures the deviation from the perfect sphere [84]. Besides, we can see that the shape of the chromosome with the most significant expanding structure is geometrically different from the one in the ES cell. However, the chromosome at the stage “C4” in cancerization is closer to the ES cell than the stage “R4” in reversion.

The chromosome structural expansion during the cancerization and reversion can also be reflected by the local and non-local contact formations (Fig 3C and 3D and S4 Fig). Here we used the fraction of “native” contact *Q*, which was widely applied in protein folding [85], with references being the averages of the pairwise distances between loci in chromosome ensembles at the normal and cancer cells. There are different pathways for local chromosome structural formations during these two processes, which appear to deviate significantly from the chromosome in the ES cell. For non-local contacts, the pathways become overlapped but still show differences at the most expanding chromosome structure. Further projections of the trajectories onto the contact probability formed by different genomic distances show simultaneous adaptations of the contacts at both local and non-local ranges (S5 Fig). These results imply that the highly irreversible chromosome structural transitions occur universally. The chromosomes undergo isotropic structural expansions followed by the compaction with adapting the shapes and the contact formations during the cancerization and reversion.

### Spatial rearrangements of chromosomal loci during cancerization and reversion

**Fig 4:**
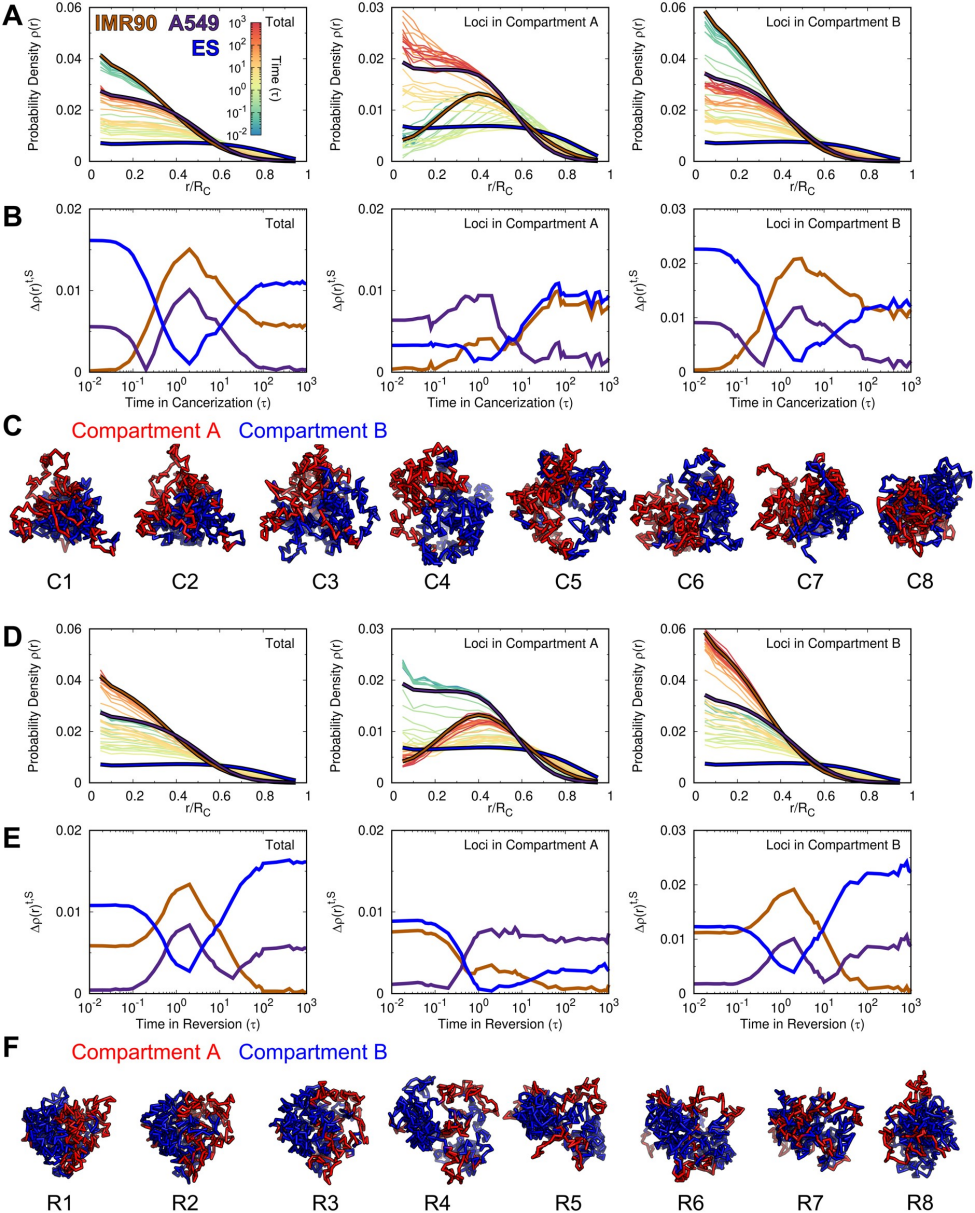
The change of the radial density in the chromosome during cell cancerization and reversion. (A) The change of the radial density profile *ρ*(*r*) for the whole loci (*Left*), loci in compartment A (*Middle*) and loci in compartment B (*Right*) in the chromosome during cancerization. The profiles of *ρ* at the IMR90, A549 and ES cells are colored brown, purple and blue, respectively. (B) The difference of the radial density, calculated by $\Delta \rho {\left(r\right)}^{t,S}=\int_{0}^{R_{C}}\left|\rho {\left(r\right)}^{t}-\rho {\left(r\right)}^{S}\right|dr/R_{C}$, from the processing time (*t*) to the reference cell (*S*). (C) Structural illustrations of the chromosome with loci colored by compartment states during cancerization. (D-F) are the same with (A-C) but for the reversion process.

It has been recognized that the spatial distribution of the chromosomal loci strongly influences the transcriptional activity [86, 87]. To see how the chromosome rearranges the spatial distribution of the chromosomal loci along with the compartment formation during cancerization and reversion, we calculated the radial density *ρ*(*r*) of chromosomal loci and further classified it into the compartment A and B. We found that the normal cell, which is a terminally differentiated cell, tends to locate the chromosomal loci of compartment A and B towards the chromosome’s surface and interior, respectively (Fig 4A). The finding is in line with previous simulations [49, 88, 89] and experiment [90]. In contrast, the chromosome in the ES cell exhibits a roughly uniform distribution of the chromosomal loci regardless of the compartment status. For the cancer cell, *ρ*(*r*) appears to be the intermediate between those at the normal and ES cells. We further calculated the radial density similarity Δ*ρ*(*r*)^*t*,*S*^ between the processing state at time point *t* and the normal, cancer and ES cells for the total loci and the loci in compartment A and B (Fig 4B). When the normal cell converts to the cancer cell, Δ*ρ*(*r*)^*t*,*S*^ of the ES cell decreases and reaches the minimum at time ∼ 0.9–3*τ*, which are varied by the total loci and the loci in the compartment A and B (Fig 4B). We note that this period corresponds to the stage when chromosome forms more expanding structure than those at the normal and cancer cell (Fig 2B), so the results indicate that the spatial distribution of the loci in chromosome during this period is similar to that in the ES cell. Further proceeding cancerization decreases Δ*ρ*(*r*)^*t*,*S*^ of the cancer cell. Overall, we found a non-monotonic spatial repositioning of the chromosomal loci during cancerization. In the beginning, the chromosome segregates the active loci located in compartment A and inactive loci located in compartment B towards the surface and interior, respectively (Fig 4C). Then, the compartment segregation of the chromosome is still effective, but it forms a stem-like pattern with a uniform radial distribution of genomic loci regardless of the compartment states. Finally, the spatial distribution of the genomic loci reaches the one at the cancer cell.

Interestingly, we observed a similar trend of spatial repositioning of the chromosomal loci during the reversion process (Fig 4D, 4E and 4F). In other words, *ρ*(*r*) during reversion also goes through processing states sharing similarity with that in the ES cell. In detail, we found that Δ*ρ*(*r*)^*t*,*S*^ of the ES cell decreases more for the loci in compartment A than the ones in compartment B at time ∼ 1–3*τ* (Fig 4E), corresponding to the stage when chromosome forms more expanding structures than those at the normal and cancer cells (Fig 2F). This is different from that observed in the cancerization process, where spatial repositioning of the chromosomal loci in compartment B is dominant to be the stem-like, as decreasing Δ*ρ*(*r*)^*t*,*S*^ of the ES is more significant for the loci in compartment B than the ones in compartment A.

In order to see how the spatial distribution of the genes on the chromosome changes during the cancerization and reversion processes, we further calculated the radial density of genes *ρ*(*r*)^*gene*^ during the transition (S6 Fig). We found that *ρ*(*r*)^*gene*^ at the normal, cancer and ES cells show similar distribution behaviors with *ρ*(*r*) of the chromosomal loci with the compartment A status at the corresponding cells. Changes in *ρ*(*r*)^*gene*^ also follow similar trends of the changes in *ρ*(*r*) of chromosomal loci with the compartment A status during both cancerization and reversion processes. The findings are likely due to the fact that the chromosomal loci in compartment A has a much higher gene density than the ones in compartment B. The combined results suggest that during both cancerization and reversion processes, the chromosome dynamically rearranges the spatial positions of the chromosomal loci and genes, and the distribution can resemble the one at the ES cell.

### Stemness and irreversibility of chromosome structural transitions during cancerization and reversion

Our results have implied that the chromosome during the cancerization and reversion may adopt the chromosome structure in the ES cell. To quantitatively assess this, we divided the contacts by different ranges and then performed the principal component analysis (PCA) on the contact probability evolving trajectories. Since the contacts at long-ranges are usually formed with very low probabilities, it may introduce large uncertainty and imprecision when directly applying to PCA. To resolve this issue, we instead used the matrix *P*_*obs*_/*P*_*exp*_, which captures the essence of the compartment formation [15]. We also note that the boundaries of TADs are mostly conserved during the transitions (S7 Fig). This indicates that the chromosome structural arrangements during cancerization and reversion should mostly rely on the contacts at the long ranges.

**Fig 5:**
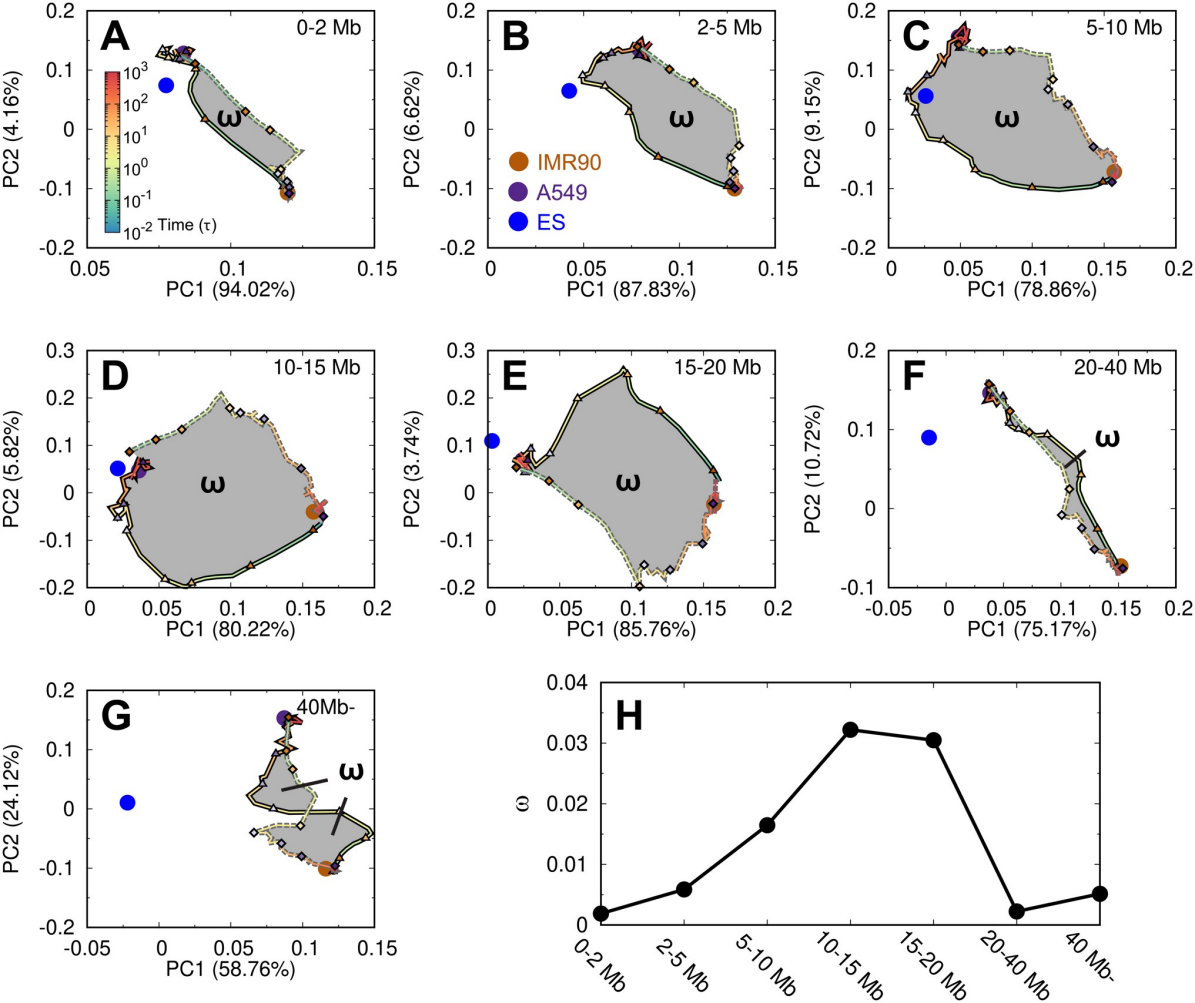
The pathways of the chromosome structural transition during cell cancerization and reversion. The PCA plots on the contact matrix *P*_*obs*_/*P*_*exp*_ evolving with the time during the processes projected on the first two PCs. The plots are further divided into different contact ranges (A-G). The reference points from the IMR90, A549 and ES cells are plotted as brown, purple and blue points, respectively. The 8 stages during the cancerization paths (solid lines) and reversion paths (dashed lines) are indicated by the triangles and diamonds, respectively. The hysteresis loop (*ω*) is colored grey in (A-G), and the area is calculated for different contact ranges (H).

We performed the PCA plots on the transition trajectories with the first and second most weighted PCs (Fig 5). In this respect, at the local range (< 2 Mb), we see that the ES cell is not on either of the two pathways. As the contact range increases (2–5 and 5–10 Mb), the location of the ES cell becomes close to the cancerization pathway but is far from the reversion pathway. Further increasing the contact range to 10–15 and 15–20 Mb shows that the ES cell is close to the cancer cell. When the contact range is very long (20–40 Mb and > 40 Mb), the ES cell locates far from both of these two pathways. Our results showed that the chromosome during the cancerization process may explore the structures formed in the ES cell at the moderate contact range (2–20 Mb). In contrast, the chromosome during the reversion process seems to be structurally different from the one in the ES cell, though the chromosome is prone to adopt many of the structural properties in the ES cell, such as the contact probability scaling, structural shape geometry and spatial distribution of the chromosomal loci.

Interestingly, we found that the two pathways do not overlap, resulting in the hysteresis phenomenon. Hysteresis indicates a toggle-like switching behavior and was often described in the context of ferromagnetism. In biology, hysteresis was found in the cell cycle and demonstrated as the driving force for the irreversible cell cycle transitions [91, 92]. In this respect, the hysteresis loop area provides a quantitative measure of the degree of the irreversibility, thus it was calculated here (Fig 5H). We found that the largest hysteresis area, which corresponds to the most significant irreversibility of the chromosome structural transitions during cancerization and reversion, is at the moderate contact range. Combining the results, we showed that the chromosome structural transitions during the cancerization and reversion processes are highly irreversible, and the chromosome tends to form stem-like structures during the cancerization.

## Discussion

We used the landscape-switching model to explore the chromosome structural transitions during the cell cancerization and reversion processes. More than two hundred independent simulations (226 simulations for cancerization and 246 simulations for reversion, see “Materials and methods”) were performed for a long time under the potential designated for describing the chromosome dynamics in the normal (or cancer) cell state. We found that these simulations with the initial chromosome structures chosen based on the clustering method recapitulate the structural ensembles in terms of the contact maps, TADs and compartments (S8 Fig). The feature suggests that the selected chromosome structures can represent the chromosome structural distributions at the original cell states and are statistically sufficient to initialize the landscape-switching simulations. The Langevin stochastic simulations were performed to study the chromosome structural dynamics with friction and noisy terms implicitly representing the hydrodynamic interactions from the surrounding environments and fluctuations from the numerous biological activities, respectively [49]. We further described the cell state transition processes between the normal and cancer cells from the chromosome structural perspective, based on the intimate relationship between the gene expression and the chromosome structure. By projecting the transition trajectories onto different order parameters, we observed highly fluctuating, stochastic and diverse pathways. The results are reminiscent of recent increasing experimental evidence that chromosome structures exhibit high cell-to-cell variability [43, 93–96]. At the same time, we found that the averaged pathways showed clear routes for the chromosomes to accomplish the structural transitions, suggesting the deterministic dynamics in organizing the chromosome structures during the cancerization and reversion. This also resonates with the single-cell Hi-C measurements on the cell cycle process, where the cyclic chromosome structural dynamics are heterogeneous at the single-cell level but show deterministic trends collectively [38]. Therefore, our simulations indicate that the chromosome structural dynamics during the cancerization and reversion are a combination of stochastic and deterministic dynamics.

In combination with the results on the contact probability decaying with the genomic distance, the global structural geometry, the contact formation and the spatial distribution of chromosomal loci, we showed that the chromosomes with the highest degree of structural expansion during cell cancerization and reversion share significant structural similarities to the ones at the ES cell. However, there are differences between the chromosomes with expanding structures during these two processes. As shown by the enhanced contact probability evolving trajectories (Fig 5), the chromosome during cancerization can form a stem-like chromosomal contact pattern up to the range of 20 Mb. In contrast, this is not observed during reversion. The results indicate a potential role of the cell state with stemness during cancerization in guiding the process. The chromosome focused in our study is the long arm of chromosome 14. From the curves of the contact probability *P*(*l*) versus genomic distance *l* for other chromosomes in the IMR90 and A549 cells (S9 Fig), we found similar trends of *P*(*l*) for the chromosomes 1 to 14 as well as their changes between the IMR90 and A549 cells. As *P*(*l*) reflects the strengths of the chromosome interactions at different genomic distances, this feature may suggest that the results obtained in our simulations are applicable to the segments in chromosomes 1 to 14 as long as there are no chromosome abnormalities.

In order to see how the transcriptional activity changes between the normal and cancer cells, we analyzed the RNA-seq data of the IMR90, A549 and ES cells. We found that the gene expression levels of the loci across the chromosome segment focused in this study are overall quite similar for these three cells (S10A Fig). However, there are more loci with low gene expression levels in the IMR90 cell than those in the A549 and ES cell (S10B Fig), indicating that a large number of genes switch from low to high levels during the cancerization. Interestingly, we found that there is a correlation between the gene expression level changes and the compartment status switching for the cell state transition between the IMR90 and A549 cells (S10C Fig). In general, the compartment switching from B to A leads to a more significant increase of the gene expression level than the compartment switching from A to B. This is a demonstration of how the chromosome reorganizes its structure in favor of gene expression (function). In our simulations, we have shown that chromosome dynamically changes the compartment status and the spatial distribution of the chromosomal loci with different compartment status during the cancerization and reversion. Thus the dynamical adaption of transcriptional activity along the chromosome should also be expected during the processes.

**Fig 6:**
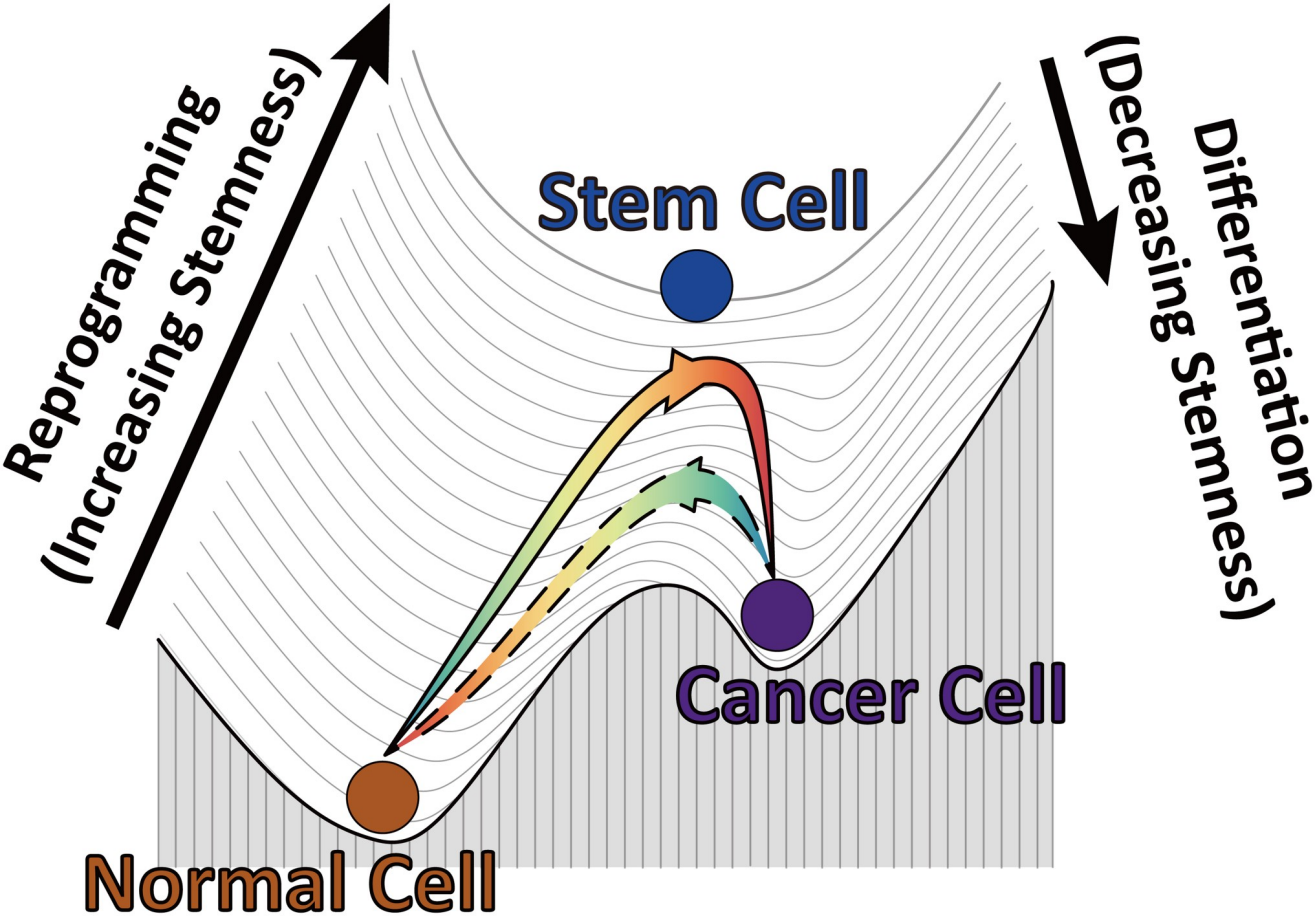
The Waddington landscape of the cell cancerization (solid line) and reversion (dashed line) processes from the chromosome structural transition perspective.

Based on our simulation results, we can propose a pictorial Waddington’s landscape from the chromosome structural perspective to understand the cancerization and reversion (Fig 6). In the context of Waddington’s epigenetic landscape [97], the cell, metaphorically referred to as a ball, rolls down from the ES cell at the top, which possesses the highest degree of stemness, to the terminally differentiated cell at the basin of the landscape to accomplish cell differentiation. As the reverse process, cell reprogramming transforms the differentiated cell to the ES cell by gaining the stemness [98, 99]. Hi-C data revealed that the chromosome at the cancer cell has more loci in compartment A than compartment B (Fig 1), corresponding to more populated active, open euchromatin than that of inactive, closed heterochromatin. The analyses on the gene expression levels at the IMR90, A549 and ES cells show an apparent increase in the transcriptional activity from the normal to cancer cells (S10 Fig). In particular, the increased gene expression levels for the chromosomal loci associated with compartment switching from B to A are more significant than those associated with compartment switching from A to B when the cell state changes from the normal to the cancer cell. These features suggest that the cancer cell, which exhibits the characteristics of the stem cell, should be located at a landscape layer, higher than the normal cell. The normal cell is terminally differentiated with a large proportion of the chromosomal loci in the repressive compartment B associated with low gene expression levels. Transforming the normal cell to the cancer cell increases the stemness. However, our simulations showed that cancerization is a non-monotonic process, during which the chromosome with the highest degree of structural expansion exhibits significant structural similarity with the one at the ES cell. In the landscape view, the cell at the first stage of cancerization climbs up on the landscape approaching the ES cell, reminiscent of cell reprogramming. The second stage of cell cancerization corresponds to a rolling down process toward the cancer cell, reminiscent of cell differentiation. On the other hand, the chromosome during the reversion process also expands and reorganizes its structure towards the one at the ES as an implication of gaining the stemness. However, the structural difference of the chromosome to the one at the ES during reversion is more significant than that during the cancerization. This leads to a relatively weak reprogramming process at the first stage of the reversion process. Thus the reversion pathway should appear to be under the cancerization pathway (Fig 6).

Recently, the non-monotonic chromosome structural transition was observed in a short-lived intermediate state during the mitotic exit process by the time-course Hi-C experiments [44]. The chromosome in this intermediate state was further characterized to be unbound with the structural maintenance of chromosomes (SMC) protein complexes, corresponding to the over-expanding chromosome observed in the simulations [53]. The advantage of the chromosome being over-expanding is likely because the large accessible surface in the loosely formed chromosome structure can facilitate the subsequent proteins binding. Besides, using an integrative approach combined with the measurements on gene expressions and epigenetic information, Cacchiarelli et al. found that reprogramming of the fibroblast cell to the stem cell is a non-monotonic process, which contains a reverse of differentiation process to the pre-implantation-like cell state followed by a transition to the post-implantation-like stem cell state at the late stage of reprogramming [100]. In light of the intimate relations among the gene expression, epigenetics and 3D genome architecture [3–5], the non-monotonic transitions in chromosome structure should also be expected during the reprogramming. With a landscape-switching model, we observed the over-expanding chromosome structures, which are more open than those at the fibroblast and ES cells, during the reprogramming, resonating with the experimental evidence [45]. Here, we found that the chromosome during cell cancerization opens its structure and partially forms the structure, similar to the one formed in the ES cell. The ES cells that are richer in the less compact euchromatin than the highly condensed heterochromatin [78], were found to form the globally open chromosome structures [101, 102]. Such open chromosome structures in the ES cell may promote the chromatin-protein binding, which controls the pluripotent differentiation [103, 104]. Our findings echo with a recent experiment using super-resolution imaging techniques [105], where Xu et al. observed that the chromosome tends to open its structure with decompaction of heterochromatin in the early stages of carcinogenesis. Furthermore, it was found that the disruption of the compact heterochromatin prior to tumor formation occurs ubiquitously across multiple cancer types and shares the similar trait of the chromosome organization with the stem cells [106].

We can propose a possible explanation for the non-monotonic chromosome structural transition during the cell state transitions observed in our model. It is worth noting that the non-monotonic chromosome structures were only observed during the transitions, where the chromosomes are more compacted in the initial state than in the final state. Compared to the less compacted chromosome, the more compacted chromosome is formed by more chromosomal contacts, associated with a smaller configurational space of the structural ensemble. During the cell state transition involving the chromosome decompaction, the contacts have to be broken to overcome the entropic barrier with the aid of the extra energy input. The breaking of the contacts led by the extra energy input can be excessive to induce over-expanding chromosome structures, which are in favor of the entropy. This provides opportunities for the system to escape the trapped state and enough configurational space associated with high energy to search for the new state. In contrast, during the cell state transitions involving the chromosome compaction, the chromosome may not have to break a significant amount of contacts. In this respect, the extra energy input tends to mainly guide the chromosome to form and stabilize the contacts to compact the chromosome, so the over-expanding chromosome structures were not observed. When it comes to the cancer processes, where the chromosomes have a similar degree of compaction at the initial and final states, the breaking and formation of contacts can occur simultaneously at comparable degrees, so the over-expanding chromosome structures can still be observed.

Our simulation showed that cancerization and reversion are irreversible processes. Previously, it was assumed that the forward and reverse processes of cell state transitions may pass through the same intermediate cell states, referred to as the metastable attractors on the Waddington’s landscape [107, 108]. Theoretical studies at the gene network level have shown that irreversibility is a universal feature for cell state transition due to the nonequilibrium effects [56, 109]. Our simulation results provided a molecular-level description that the irreversibility of cell cancerization and reversion can be reflected by the chromosome structural dynamics. Besides, we proposed a quantitative way to measure the irreversibility in cancerization and reversion using the hysteresis loop analysis of the chromosomal contact formation. Our findings also resonate with a recent experiment, which used a combination of Hi-C and replication timing analyses to characterize the irreversible non-overlapped pathways between the cell state transition, namely the differentiation and reprogramming [110].

The stemness-related genetic markers and interactions in cells are controlled by the underlying gene regulatory network [111], which uses chromosomes as the structural scaffold for functionalities. Besides, the CS cells were found to have similar marker expression profiles with normal stem cells [112]. These features support that our approach for inferring the stemness and connecting it to the partial formation of the CS cell is reasonable and based on the intimate structure-function relationship at the genomic level [4, 5, 86]. Therefore, we speculate that the stem-like intermediate cell state during cell cancerization may contribute to the formation of the CS cells, of which the terminally differentiated somatic cell has been suggested as one potential origin [113]. Due to the lack of the Hi-C data at the CS cells, the precise and direct comparisons of the intermediate state to the CS cells were not performed. A strict assessment of our statement can be made when the experimental data are available.

Our predictions of the transient intermediate state with stemness during cell cancerization may have implications for cancer treatments. The increase of stemness during cancerization may promote the cell self-renewal and differentiation processes, similar to what the CS cells do. Thus, decreasing the stemness of the cell by deviating the chromosome structures from the one at the ES cell can help to repress the cancer initiation and progression. Further efforts can be focused on developing effective genome structure engineering methods for modulating and disrupting the specific chromosomal interactions similar to those formed in the ES cells [114–118], serving as a potential therapeutic approach.

Our predictions can be tested by future experiments. In this regard, the time-course Hi-C experiments appear to be promising and competent in monitoring the chromosome structural evolution during the cancerization and reversion processes [37]. It is expected that the Hi-C data at the high temporal resolution can provide a dynamical picture of how the chromosome structure rearranges and offer useful clues as to the formation of the stem-like intermediate state during cell state transition using advanced data-analysis methods [44, 119]. Meanwhile, mounting theoretical and experimental studies confirm the existence of intermediates during the epithelial-mesenchymal transition (EMT) [120–123], which contributes to cancer metastasis and tumor relapse. The EMT intermediates often exhibit a gain of stemness compared with the epithelial and mesenchymal cells [124–127], leading to a non-monotonic increasing-followed-by-decreasing trend in stemness during the EMT [128]. These properties of the EMT process are similar to what we have observed in our simulations of the transition from the normal cell to the cancer cell. Therefore, the fruitful approaches developed for uncovering the EMT intermediate and studying the EMT pathways can be adapted to investigate the cancerization processes [129]. Ultimately, the combined efforts on the direct cancerization and the EMT processes will help us to complete the understanding of how cancer initializes and metastasizes.

Our landscape-switching model can be improved from the following three aspects. First, the model used a simplified regime at the nonadiabatic limit to describe the cancerization and reversion processes. However, the ratio between the rates of the intra- and inter-landscape dynamics are not infinite, thus there is a certain degree of adiabaticity in the cell state transitions processes. To take into account the adiabaticity, more switches of the landscape can be implemented, which can result in an elevated rate for the inter-landscape hopping [66, 67]. In this regard, the experimental determinations of the timescales for cancer initiation and progression are prerequisites to include the adiabaticity in the model. Noteworthy, as an extreme case where multiple switches of the landscape occur frequently, corresponding to a much faster inter-landscape hopping than intra-landscape motion, the process approaches the adiabatic limit, which can be described by an averaged classic intra-landscape barrier crossing on a single effective landscape [130]. In practice, cell cancerization and reversion should occur between two extreme cases: the nonadiabatic landscape-switching process and the effective intra-landscape barrier-crossing process. The precise determination of the adiabaticity of the process relies on future experiments focusing on measuring the timescales of the chromosome dynamics relaxation within one cell state and hopping between the two cell states. Second, the model considers a bistable system with abrupt switches for describing the cell state transitions. However, the cell state transitions should occur continuously with finite rates in reality. This indicates that further improvements on the connections between these two landscapes are needed. Nevertheless, there is still a lack of clear guidance to determine the form of the connections that can best capture the essence of the processes. One may build a “double-basin” landscape widely used in studying the protein conformational changes, which usually occur between the two distinct structural states [131–133]. However, this landscape leads to an equilibrium process with an often very high potential barrier, which makes it almost impossible to switch the state. This contradicts the nonequilibrium nature of chromosome structural changes during the cell state transitions, which require significant energy input to help overcome the barrier. The landscape-switching model used a simple way to establish the connections between two landscapes with considering the nonequilibrium effects. Further improvements on the model can be made by refining the form of the connections between the two landscapes with the experiments. It is worth noting that the (meta)stable intermediate cell states may exist during the cancerization and reversion [134]. Thus, we expect that including the intermediate states through stepwise switches among the multiple (meta)stable cell states in the system can improve the accuracy of the connections between the normal and cancer cells. This can be implemented in future studies when the experimental Hi-C data on the intermediate states are available. Third, the model did not take into account the cell cycle process. The cell state transitions between the normal and cancer cells often undergo multiple rounds of cell cycle processes. In general, the interphase occupies a significant amount of time during the cell cycle. In this regard, we focused on the slow cell state transitions in the interphase during the cell cancerization and reversion processes while treating the fast cell cycle dynamics as an averaged background. In reality, the cell development and cell cycle are controlled by two different regulatory networks [135]. The quantitative characterization of the interplay between these two networks in future studies can help the further improvements on the model by incorporating the cell cycle processes.

In summary, we presented a chromosome structural-level study of illuminating the molecular mechanisms of cancer formation and its reversion processes. The targeted cells belong to the human lung organism and should be regarded as one example of cancer. Up to now, more than a hundred distinct types of cancer have been uncovered. Different types of cancer may vary substantially in their behaviors. However, all types of cancer are caused by the common mechanism of uncontrolled cell division, reflecting the dysregulation of cell self-renewal. Therefore, we speculate that our observation of the stem-like intermediate state during cancer formation may likely be observed by the other types of cancer. The open chromosome structure in the stem-like intermediate state may contribute to the increased genomic instability and active transcription for cells to gain plasticity that has been demonstrated as the universal key to facilitate cancer progression [136]. Future studies on elucidating the cancer formation pathways and the stem-like intermediate state can help to decipher the fundamental mechanisms that underlie the different types of cancer.

## Materials and methods

### Chromosome polymer model

We built a beads-on-a-string model at the 100 kb resolution to represent the chromosome segment (chr14: 20.5–106.1 Mb), resulting in a total of 857 beads in the system. The basic background of our polymer model is a generic polymer model with homogeneously weighted bonded and nonbonded potentials:

$$\begin{array}{c} V_{\text{Polymer}}=V_{\text{Bond}}+V_{\text{Nonbond}}. \end{array}$$

The bonded potential contains the finitely extensible nonlinear elastic (FENE) bond stretching interactions, linear-promoting angle bending interactions, and neighboring beads non-overlapped interactions with the expression as follows [137]:

$$\begin{array}{c} V_{\text{Bond}}=V_{\text{FENE}}+V_{\text{Angle}}+V_{\text{hc}}, \end{array}$$

where the FENE potential for the bond (*r*_*i*,*i*+1_) between neighboring beads (i, i+1) is expressed as:

$$\begin{array}{c} V_{\text{FENE}}=-0.5K_{b}R_{0}^{2}\;\text{ln}\left[1-{\left(\frac{r_{i,i+1}}{R_{0}}\right)}^{2}\right], \end{array}$$

and the non-overlapped interaction between (i, i+1) is described by a hard-core Lennard-Jones potential (*V*_*LJ*_):

$$\begin{array}{c} V_{hc}=\{\begin{array}{cc} V_{LJ}\left(r_{i,i+1}\right), & r_{i,i+1}\leq \sigma 2^{1/6},\\ 0, & r_{i,i+1}>\sigma 2^{1/6}, \end{array} \end{array}$$

where

$$\begin{array}{c} V_{LJ}\left(r_{i,j}\right)=4\epsilon [(\frac{\sigma }{r_{i,j}}{)}^{12}-(\frac{\sigma }{r_{i,j}}{)}^{6}]+\epsilon . \end{array}$$

To consider the stiffness of the chain at the local range, we applied an angle potential acting on the three adjacent beads (*i* − 1, *i*, *i* + 1) with the following form, which was used in the previous studies [72, 137]:

$$\begin{array}{c} V_{\text{Angle}}=K_{a}\left[1-\text{cos}\left(\theta_{i}-\pi \right)\right]. \end{array}$$

The angle term describes the local stiffness of the chain, thus it favors the linear placement of the three adjacent beads with *θ*_*i*_ = *π*.

The nonbonded potential for every nonbonded pair (i,j) was set as the soft-core interactions that allow the chain-crossing to mimic the effects of a large number of topoisomerases in the cell nucleus on efficiently unknotting the DNA molecules *in vivo* [138, 139].

$$\begin{array}{c} V_{\text{Nonbond}}=\{\begin{array}{cc} 2(1+\text{tanh}[0.5V_{LJ}\left(r_{i,j}\right)-1]), & r\leq r_{0},\\ V_{LJ}\left(r_{i,j}\right), & r_{0}<r_{i,j}\leq \sigma 2^{1/6},\\ 0, & r_{i,j}>\sigma 2^{1/6}. \end{array} \end{array}$$

In addition, a spherical confinement with the radius *R*_*C*_ was used to mimic the volume fraction of the chromosome in the cell nucleus at 10%, same as used previously [72, 137]. With only the generic polymer potential, the model generates an ensemble that resembles the equilibrium globule [45].

Reduced units were used throughout simulations. The energy unit was *ϵ* = 1.0. The bond length *σ* was set to be the length unit. In FENE potential, *R*_0_ = 1.5*σ* allows the bonds to stretch flexibly and *K*_*b*_ = 30.0/*σ*^2^. The angle potential has a moderate strength of *K*_*a*_ = 2.0. We note that a very large *K*_*a*_ would lead to a high degree of chain stiffness at the local range, contradictory to the highly flexible nature of the chromatin. In contrast, a very weak angle term would eliminate the effects of the stiffness. In the soft-core potential, we set $r_{0}=\sigma /{\left(\left(1+\sqrt{2}\right)/2\right)}^{1/6}$, so *V*_*LJ*_(*r*_0_) = 2.0 [72]. The radius of the spherical confinement *R*_*C*_ was set to be 9.7*σ*. The temperature in all simulations was set to 1.0 in energy units by multiplying by the Boltzmann constant. Langevin stochastic dynamics was applied with a time step of 0.0005*τ* and a friction coefficient of 10.0*τ*^−1^, where *τ* is the reduced time unit.

Growing lines of evidence showed that the activities of topoisomerases are effective throughout the cell cycle [140–142]. For simplicity, we kept the soft-core potential constant throughout the simulations. The strength of *V*_Nonbond_ appears to be moderate with a maximum of 4.0 when the two beads are fully overlapped. A very strong soft-core potential, which resembles the hard-core potential, would reduce the possibility of chain-crossing [143]. In contrast, a very weak soft-core potential could lead to an unrealistic biological picture, where the chromosomal loci do not have excluded volumes and the reaction rates of the topoisomerases on DNA topology are infinite.

### Maximum entropy principle simulation

The potential *V*(***r***) in the maximum entropy principle simulations is made up of the generic polymer potential *V*_Polymer_ and the Hi-C restraint potential *V*_Hi-C_:

$$\begin{array}{c} V\left(r\right)=V_{\text{Polymer}}+V_{\text{Hi-C}}. \end{array}$$

The entropy of system under *V*(***r***) relative to a given prior distribution *ϱ*_0_(***r***) under polymer potential *V*_Polymer_ is defined as [71]:

$$\begin{array}{c} S[\varrho ||\varrho_{0}]=-\int dr\varrho \left(r\right)ln\frac{\varrho (r)}{\varrho_{0}\left(r\right)}. \end{array}$$

This entropy should be maximized subject to the following constraints in order to be compatible with observations (contact probability *P*_*i*,*j*_ and experimental Hi-C data *f*_*i*,*j*_):

$$\begin{array}{c} \left\{ \begin{array}{cc} & \int dr\varrho \left(r\right)P_{i,j}=\left\langle P_{i,j}\right\rangle =f_{i,j},\\ & \int dr\varrho (r)=1. \end{array}\right. \end{array}$$

The maximization of the entropy can be obtained using the method of Lagrangian multipliers by searching for the stationary points of the Lagrange function:

$$\begin{array}{c} L=S[\varrho ||\varrho_{0}]+\sum_{i,j}\alpha_{i,j}\left(dr\varrho \left(r\right)P_{i,j}-f_{i,j}\right)+\beta \left(\int dr\varrho \left(r\right)-1\right), \end{array}$$

where *α*_*i*,*j*_ and *β* are Lagrangian multipliers. By setting $\frac{\delta L}{\delta \varrho (r)}=0$ and neglecting the normalization factor, the posterior distribution has the following expression:

$$\begin{array}{c} \varrho \left(r\right)\propto e^{-\sum_{i,j}\alpha_{i,j}P_{i,j}}\cdot \varrho_{0}\left(r\right). \end{array}$$

Therefore, the potential *V*(***r***) with maximum entropy principle, is expressed as follows:

$$\begin{array}{c} V\left(r|S\right)=V_{\text{Polymer}}+\sum_{i,j}\alpha_{i,j}P_{i,j}, \end{array}$$

where *V*_Hi-C_ is in a linear form of contact probabilities and *S* represents the cell state (IMR90 or A549). In practice, *P*_*i*,*j*_ is the calculated contact probability between the chromosomal loci *i* and *j* using step function expressed as:

$$\begin{array}{c} P_{i,j}=\frac{1}{2}\left[1+tanh\left(\mu \left(R_{0}-r_{i,j}\right)\right)\right], \end{array}$$

where we set *μ* = 3.0/*σ*. *α*_*i*,*j*_ is the corresponding contact strength, which is iteratively adapted by simulations. *V*_Hi-C_ acts on the non-neighboring beads with |*i* − *j*| > 1, which includes the pair (*i* − 1, *i* + 1) involved in the angle term. During the iteration process, the contact probability *P*_*i*,*j*_ is restrained by the Hi-C data *f*_*i*,*j*_. In the end, the chromosome under the potential *V*(***r***|*S*) generates an ensemble that can reproduce the Hi-C maps at the normal and cancer cell, separately (S1 Fig). Details of the maximum entropy principle simulation can be found in previous studies [72, 73]. The maximum entropy principles simulations for the ES and IMR90 cells were done in our previous work [45].

### Landscape-switching model

We used the resulting potentials *V*(***r***|*S*) of the maximum entropy principle simulations for the normal and cancer cells to represent the effective energy landscapes. We performed the hierarchical clustering on the chromosome ensembles generated by the maximum entropy principle simulations. Two chromosome structures in each cluster, which has a population higher than 0.3%, were picked out as the initial structures for performing the landscape-switching simulations (S2 Fig). The purpose of choosing the chromosome structures in the populated clusters rather than using the whole sets of the structures in the ensembles is to generate a limited number of structures that can sufficiently represent the ensemble in order to significantly reduce the computational expenses. This led to 226 and 246 trajectories for the cancerization and reversion processes, respectively.

The landscape-switching simulations were performed as follows. First, the simulations were run under the potential *V*(***r***|*IMR*90) (cancerization) or *V*(***r***|*A*549) (reversion) for 5000*τ*, where *τ* is the time unit of the simulations. Then a sudden switch of potential in terms of *V*(***r***|*IMR*90) → *V*(***r***|*A*549) (cancerization) or *V*(***r***|*A*549) → *V*(***r***|*IMR*90) (reversion) was implemented. Finally, the simulations were under the new potential *V*(***r***|*A*549) (cancerizatin) or *V*(***r***|*IMR*90) (reversion) for 10000*τ*. All the trajectories were collected and combined for the analyses to generate the results present in this study.

### Trajectory analysis

To cluster the processing states during the transition based on the chromosome structural similarity, we calculated the contact map difference Δ*P*^*I*,*J*^ between the time point *t* = *I* and *t* = *J* by:

$$\begin{array}{c} \Delta P^{I,J}=\sum_{i,j}\left|P_{i,j}^{t=I}-P_{i,j}^{t=J}\right|/\sum_{i,j}P_{i,j}^{t=I}, \end{array}$$

where $P_{i,j}^{t=I(J)}$ is the contact probability between the chromosomal loci *i* and *j* at the time *t* = *I* or *J* during the transition. Δ*P*^*I*,*J*^ was then normalized from 0 to 1. We performed the hierarchical clustering on Δ*P*^*I*,*J*^ and presented the dendrogram on the relationships between the processing states during the transition. This was done based on the total contact pairs, local contact pairs (|*i* − *j*| < 2*Mb*) and non-local contact pairs (|*i* − *j*| ≥ 2*Mb*). In order to see how the chromosome structures evolve with respect to the ones of the IMR90, A549 and ES cells during the transitions, we replaced the contact probability map at the time point *t* = *J* with the ones at the IMR90, A549 and ES cells. Therefore, we obtained the differences of contact maps at the processing states relative to the Hi-C data of the IMR90, A549 and ES cells:

$$\begin{array}{c} \Delta P^{t,S}=\sum_{i,j}\left|P_{i,j}^{t}-P_{i,j}^{S}\right|/\sum_{i,j}P_{i,j}^{t}, \end{array}$$

where *S* stands for the IMR90, A549 and ES cells, respectively.

The fraction of native contacts *Q* was used to compare the structural similarity of the chromosomes to ones at the IMR90 or A549 cells. The references of the pairwise distances in *Q* were set to be the averages of the pairwise distances in the chromosome ensemble at the IMR90 or A549 cell obtained from the maximum entropy principle simulations. The fraction of native contacts *Q*_*i*,*j*_ for the chromosomal loci *i* and *j* is expressed as:

$$\begin{array}{c} Q_{i,j}^{IMR90(A549)}=\frac{1}{N_{sum}}\sum_{i,j}exp\left[-\frac{{\left(r_{i,j}-\left\langle r_{i,j}^{IMR90(A549)}\right\rangle \right)}^{2}}{2{\left(0.5\sigma \right)}^{2}}\right], \end{array}$$

where *N*_*sum*_ is the number of the summed pairs and $\langle r_{i,j}^{IMR90(A549)}\rangle$ is the averaged distance between the chromosomal loci *i* and *j* of the structural ensemble at the IMR90 (A549) cell. To see how the chromosome structures form at different genomic sequence distances, *Q* can be calculated based on the genomic distance *l*:

$$\begin{array}{c} Q_{l}=\frac{1}{N_{sum}}\sum_{i,j}^{|i-j|\in l}Q_{i,j}. \end{array}$$

The radial density *ρ*(*r*) was used to describe the spatial distribution of chromosomal loci, expressed as:

$$\begin{array}{c} \rho \left(r\right)=\frac{1}{N}\frac{n(r)}{4\pi r^{2}\Delta r}, \end{array}$$

where *N* is the number of the beads in the simulation, *r* is the distance to the center of the spherical confinement, which has a radius of *R*_*C*_, *n*(*r*) is the number of chromosomal loci found in the spherical shell of *r*, *r* + Δ*r*. Here, we set Δ*r* = *R*_*C*_/20. We further calculated *ρ*(*r*) based on the loci from different compartment status and gene density, which gives rise to the radial gene density *ρ*(*r*)^*gene*^.

We calculated the enhanced contact matrix *P*_*obs*_/*P*_*exp*_, which is the ratio between the observed contact probability *P*_*obs*_ and expected contact probability *P*_*exp*_ [15]. The matrix was then divided into different categories based on different genomic distances *l* between the interacting loci. We performed the PCA plots of the time evolution of the *P*_*obs*_/*P*_*exp*_ at different genomic distances *l* during the transitions. In practice, a PCA plot shows clusters of samples based on their similarity and reduces the number of dimensions by constructing PCs. The first two PCs were shown to describe the trajectories of *P*_*obs*_/*P*_*exp*_ evolution.

## Supporting information

S1 Text Additional materials and methods. (PDF)

S1 Fig Comparisons of the maximum entropy principle simulation results and the experimental Hi-C data in the A549 cells. (A) Hi-C contact maps of the chromosome ensemble obtained from the simulations and the Hi-C data at the global (*Left*) and local (*Right*) scales. (B) Contact probability versus genomic distance in the chromosome for the simulations and the Hi-C data with a slope of -1.0 in the logarithmic scale at 0.5–7 Mb. (C) Insulation score of the chromosome obtained by the simulations and the Hi-C data. (D) Compartment profiles of the chromosome obtained by the simulations and the Hi-C data. (E) Correlations of insulation score (*Left*) and compartment profiles (*Right*) between the simulations and the Hi-C data.
(TIF)

S2 Fig Chromosome ensemble in the A549 cells. (A) Contact distance (*d*) versus genomic distance (*l*) in the chromosome. (B) The probability distributions of aspheric parameters of the chromosome. Δ and *S*_*shape*_ were calculated using the inertia tensor [84]. Deviation of Δ from 0 (the value corresponding to a sphere) gives an indication of the extent of anisotropy. Negative values of *S*_*Shape*_ correspond to oblate shapes and positive values of *S*_*Shape*_ to prolate shapes. (C) The probability distribution of the configurational extension on the three principal axes of the chromosome. (D) The time evolution of the average root mean square distance (*d*_*rms*_) between every genomic pair in chromosome at the time *t* relative to its initial value: *δ*(*t*) = ∑_*i*,*j*_ *d*_*rms*_(*i*, *j*, *t*)/*N*_*sum*_, where *N*_*sum*_ is the number of summed pairs. The maximum of *δ*(*t*) is close to and below 3*σ*. (E) The hierarchical clustering of the chromosome structural ensemble shown as a dendrogram (*Top*) and the populations of the cluster (*Bottom*). Cut-off distance 3*σ* was applied. (F) The top 3 most populated chromosome clusters. Each is shown with a mixed contact map (*Left*), which contains 5 structures within the cluster, and one representative structure (*Right*).
(TIF)

S3 Fig Chromosome diffusion dynamics in the IMR90 and A549 cells. (A) Fitting of MSD to the power-law function (MSD ∼ *αx*^*β*^) in the IMR90 cell. MSD was calculated by averaging the MSD from 5 independent simulations with the potential from the maximum entropy principle simulation *V*(***r***|*IMR*90). (B) MSD of all the individual chromosomal loci in the IMR90 cell state obtained from one simulation with average shown as the black line. (C) and (D) are same as (A) and (B) but for the A549 cell.
(TIF)

S4 Fig Pathways of chromosome structural transitions projected on *Q* varied by different contact ranges during cancerization (*Left*) and reversion (*Middle*), as well as their averages (*Right*). (TIF)

S5 Fig Pathways of chromosome structural transitions projected on contact probability varied by different contact ranges during cancerization (*Left*) and reversion (*Middle*), as well as their averages (*Right*). (TIF)

S6 Fig Gene density and radial density distribution of the genes in the chromosome during cell cancerization and reversion. (A) Gene density along the chromosome and the compartment status of chromosomal loci at the IMR90, A549 and ES cells. Compartment A and B are colored red and blue, respectively. (B) The change of the radial gene density profile *ρ*(*r*)^*gene*^ in the chromosome during cancerization. The profiles of the IMR90, A549 and ES cells are colored brown, purple and blue, respectively. (C) The difference of the radial density from the processing time to the reference cell (the IMR90, A549 or ES cell) during cancerization. (D) and (E) are the same with (B) and (C) but for the reversion process. The plotting details of (B-E) are the same with Fig 4, but for the radial density distributions of the genes.
(TIF)

S7 FigTAD structural formation.(A-C) The correlation between the insulation score among the IMR90, A549 and ES cells. The correlation coefficient of insulation score of the processing state during (D) cancerization and (E) reversion with those of the IMR90, A549 and ES cells. (F) An illustration of TAD boundary overlapping between the Hi-C data (IMR90 or A549) and simulation processing state during cancerization and reversion. The numbers of TAD boundaries detected by insulation score for experimental Hi-C (IMR90 or A549), simulation processing state, and the overlap between the Hi-C (IMR90 or A549) and processing state are denoted as $N_{Hi-C}^{IMR90(A549)}$, *N*_*PS*_, and $N_{ol}^{IMR90(A549)}$, respectively. (G) The change of TAD boundary overlapping population from the simulation processing state to the Hi-C of the IMR90 and A549 (calculated by $N_{ol}^{IMR90}/N_{Hi-C}^{IMR90}$ and $N_{ol}^{A549}/N_{Hi-C}^{A549}$) and from the Hi-C of the IMR90 and A549 to the simulation processing state (indicated by superscript “+” and calculated by $N_{ol}^{IMR90}/N_{PS}$ and $N_{ol}^{A549}/N_{PS}$) during cancerization and reversion.(TIF)

S8 Fig The comparisons of the chromosome structures between the clusters for initializing the landscape-switching simulations and the ensemble generated by the maximum entropy principle simulations. (A) The IMR90 cell. (B) The A549 cell.
(TIF)

S9 Fig Contact probability versus genomic distance in Hi-C data for chromosomes 1-22 at the IMR90 (brown) and A549 cells (purple). The dashed lines indicate the chromosome segment used in this study (chr14: 20.5-106.1 Mb).
(TIF)

S10 Fig Gene expression at the IMR90, A549 and ES cells. (A) Gene expression level along the chromosomal loci. Gene expression data were measured by the RNA-seq. The expression level for each bead in our model, which represents a DNA segment of 100 Kb in length, was determined as the sum of the reads in this 100 kb DNA segment. The value was further scaled by 10^6^ to indicate the reads of “per million” and was represented in the logarithmic scale (*log*_2_). The calculation of the gene expression level is similar to the Reads Per Kilobase Million (RPKM), widely used in RNA-seq analysis. (B) Distributions of gene expression levels in the IMR90, A549 and ES cells. (C) Distributions of the changes in the gene expression levels for the genes that change the compartment status (“A to B” or “B to A”) or that remain the same (“stable”) when comparing the IMR90 cell to the A549 and ES cells, respectively. The red lines and green diamonds in the box plots indicate the median and mean values of the distributions, respectively.
(TIF)

S11 Fig The correlations between the contact probability from the maximum entropy principle simulations and the Hi-C data for the IMR90, A549 and ES cells. Pearson’s correlation coefficients were calculated based on the absolute values and logarithmic scaled values of contact probability. The calculations based on the logarithmic scaled values of contact probability were performed after removing the contacts with the probability equal to 0 in the Hi-C data. The linear fit of the simulated contact probability and the Hi-C data is shown.
(TIF)

S12 Fig Polymer ensemble from the simulations with the generic polymer model under the potential *V*_*Polymer*_ and spherical confinement. (A) The contact maps of the polymer at global (*top*) and local (*bottom*) scales. (B) Insulation score.
(TIF)
