# Supplementary material for: Deciphering the molecular mechanism of the cancer formation by chromosome structural dynamics
Source: PLoS Comput Biol. 2021 Nov 9;17(11):e1009596. doi: 10.1371/journal.pcbi.1009596 (PMC8631624; doi:10.1371/journal.pcbi.1009596)
Supplement: S1 Text — (PDF) [file pcbi.1009596.s001.pdf]

Supporting Information

for

Deciphering the molecular mechanism of the cancer formation by chromosome structural dynamics

Xiakun Chu[1], and Jin Wang[1,2*]

[1] Department of Chemistry,

State University of New York at Stony Brook, Stony Brook, New York, USA

[2] Department of Physics and Astronomy

State University of New York at Stony Brook, Stony Brook, New York, USA

[*] jin.wang.1@stonybrook.edu

Materials and Methods

Hi-C and RNA-seq data processing

We used the human fetal lung fibroblast cell (IMR90) and human lung carcinoma cell (A549) as the normal and cancer cells to investigate the cancerization and the reversion processes. The Hi-C data of the embryonic stem (ES) cell and IMR90 were downloaded from the publicly available Gene Expression Omnibus (GEO) repository archives with accession number GSE35156 [1] and the Hi-C data of A549 cell line was obtained from the ENCODE project [2] with GEO accession number GSE105600. All the replicas were combined and proceeded to the Hi-C Pro software following standard pipelines for generating the contact maps at a resolution of 100kb [3]. In this study, we focused on the long arm of chromosome 14 (20.5-106.1Mb), so the polymer model has 857 beads.

We obtained the gene expression profiles from the RNA-seq data, which were downloaded from the GEO repository archives with accession number GSE90263 for the IMR90 cell, GSE86657 for the A549 cell and GSE90225 for the ES cell [2]. The gene expression profiles at 100kb resolution for the chromosome 14 segment (20.5-106.1Mb) were calculated and assigned to the individual beads in our model. The value of the gene expression level was determined by the number of the reads (both plus and minus in RNA-seq data) averaged within the bead, mimicking the Reads Per Kilobase Million (RPKM), which is widely used in RNA-seq analysis. The gene density profile was based on the hg19 genome.

Maximum entropy principle simulations

We conducted maximum entropy principle simulations for generating the chromosome structural ensembles that lead to the contact probability maps resembling the Hi-C data in the IMR90, A549 and ES cells, respectively. The simulations were performed with multiple rounds of optimization iterations, eventually resulting in the potential $V(\boldsymbol{r}|S)$, referred to as the energy landscape for the cell state S, where S represents the IMR90, A549 and ES cells, respectively. To achieve sufficient sampling for each iteration in the maximum entropy principle optimization process, we performed 100 independent simulations starting from different chromosome structures for one cell state. To further improve the sampling, two stages were used in each simulation. The first stage is the simulated annealing simulation, during which the temperature was linearly decreased from 4.0 to 1.0 within 250τ. This stage starting at a high temperature and gradually declining to 1.0, can help to generate independent replicas for the next production simulation. The second stage is the production simulation with the constant temperature simulation fixed at the temperature 1.0, having a length of 750τ. We

collected the last 500τ trajectory from each simulation for analysis. To generate one single trajectory, we used 4 Xeon E5-2683v3 processors running for 5 hours. This results in a total of 2000 core hours for accomplishing one maximum entropy principle iteration. At the end of each iteration, we calculated the difference between the simulated ($P_{i,j}$) and experimental ($f_{i,j}$) contact probability maps M, defined as:

$$M = \sum_{i,j} |P_{i,j} - f_{i,j}| / \sum_{i,j} f_{i,j}$$

.

We terminated the maximum entropy principle optimization process when the calculated difference M is smaller than 10%, as suggested by Zhang and Wolynes [4, 5]. Finally, It took 37, 44 and 22 rounds of iterations in maximum entropy principle simulations for the IMR90, A549 and ES cells, respectively.

To see whether the simulated contact probability $P_{i,j}$ can well reproduce the Hi-C data $f_{i,j}$, we further calculated the Pearson's correlation coefficient between the $P_{i,j}$ and $f_{i,j}$ for the IMR90, A549 and ES cells, respectively. We found that all the correlation coefficients are high, close to 1 (≥ 0.97), confirming the validity of our simulations (S11 Fig).

Identifications of TADs and compartments

We used the insulation score proposed by Crane et al. to describe the strengths of TAD boundary formations [6]. The same size of sliding square (500 kb $\times$ 500 kb) as suggested in the original work was applied to calculate the insulation score. The valley/minimum of the insulation score profile indicates a strong local insulation tendency to form the TAD boundary. The boundaries of TADs were determined by the slope of the insulation score profile following the same protocol used in the original work [6]. We found that the average sizes of TADs for the chromosome segment focused in our study in the IMR90, A549 and ES cells are 1.01 Mb, 1.13 Mb and 0.91 Mb, respectively. As our chromosome model has a resolution at 100 kb, the average numbers of beads that form TADs in the IMR90, A549 and ES cells are about 10, 11 and 9, respectively. To see whether the model can capture the TAD formation, we performed the simulations with only potential $V_{Polymer}$ and the spherical confinement. We found that there is no TAD formed with the insulation scores equal to 0 across the whole chromosome segment (S12 Fig). In this regard, TADs can be reliably established by forming and enhancing the block-sized contacts between the non-bonded beads, which are separated by the genomic sequence distance longer than 300 kb (involving at least three beads).

The compartment profiles were calculated by the enhanced contact probability matrix P_{obs}/P_{exp}, which is the ratio between the observed contact probability P_{obs} and expected con-

tact probability P_{exp} [7]. The enhanced contact probability map was built at a resolution of 1Mb. Then the Iterative Correction and Eigenvector Decomposition (ICE) method was used to perform the normalization [8]. The principal component analysis (PCA) was performed on the normalized matrix and the first principal component (PC1) was referred to as the compartment profiles. The direction of the PC1 values is arbitrary, and we set the positive and negative signs of the PC1 in accordance with gene density (positive to the gene-rich region and negative to the gene-poor region). These direction assignments of the PC1 were done on the IMR90, A549 and ES cells.

For the states during the transition processes, we used the same calculation procedure to obtain the PC1 from the simulated contact probability for a particular time point during the transition. Then, the direction of the PC1 was assigned by minimizing the differences to the PC1 of the state at the previous time step via calculating the correlation coefficient with the positive and negative signs of the PC1. We performed this assignment for each state progressively, starting from the IMR90 cell for the cancerization process and the A549 cell for the reversion process. We applied this strategy based on the fact that the chromosome structural changes during the cancerization and reversion should be continuous.

References

[1] Dixon JR, Selvaraj S, Yue F, Kim A, Li Y, Shen Y, et al. Topological domains in mammalian genomes identified by analysis of chromatin interactions. Nature. 2012;485(7398):376.

[2] Consortium EP, et al. An integrated encyclopedia of DNA elements in the human genome. Nature. 2012;489(7414):57–74.

[3] Servant N, Varoquaux N, Lajoie BR, Viara E, Chen CJ, Vert JP, et al. HiC-Pro: an optimized and flexible pipeline for Hi-C data processing. Genome Biol. 2015;16(1):259.

[4] Zhang B, Wolynes PG. Topology, structures, and energy landscapes of human chromosomes. Proc. Natl. Acad. Sci. U.S.A. 2015;112(19):6062–6067.

[5] Zhang B, Wolynes PG. Shape transitions and chiral symmetry breaking in the energy landscape of the mitotic chromosome. Phys. Rev. Lett. 2016;116(24):248101.

[6] Crane E, Bian Q, McCord RP, Lajoie BR, Wheeler BS, Ralston EJ, et al. Condensin-driven remodelling of X chromosome topology during dosage compensation. Nature. 2015;523(7559):240.

[7] Lieberman-Aiden E, Van Berkum NL, Williams L, Imakaev M, Ragoczy T, Telling A, et al. Comprehensive mapping of long-range interactions reveals folding principles of the human genome. Science. 2009;326(5950):289–293.

[8] Imakaev M, Fudenberg G, McCord RP, Naumova N, Goloborodko A, Lajoie BR, et al. Iterative correction of Hi-C data reveals hallmarks of chromosome organization. Nat. Methods. 2012;9(10):999.
