# Supplementary figures and images for: Deciphering the molecular mechanism of the cancer formation by chromosome structural dynamics
Source: PLoS Comput Biol. 2021 Nov 9;17(11):e1009596. doi: 10.1371/journal.pcbi.1009596 (PMC8631624; doi:10.1371/journal.pcbi.1009596)

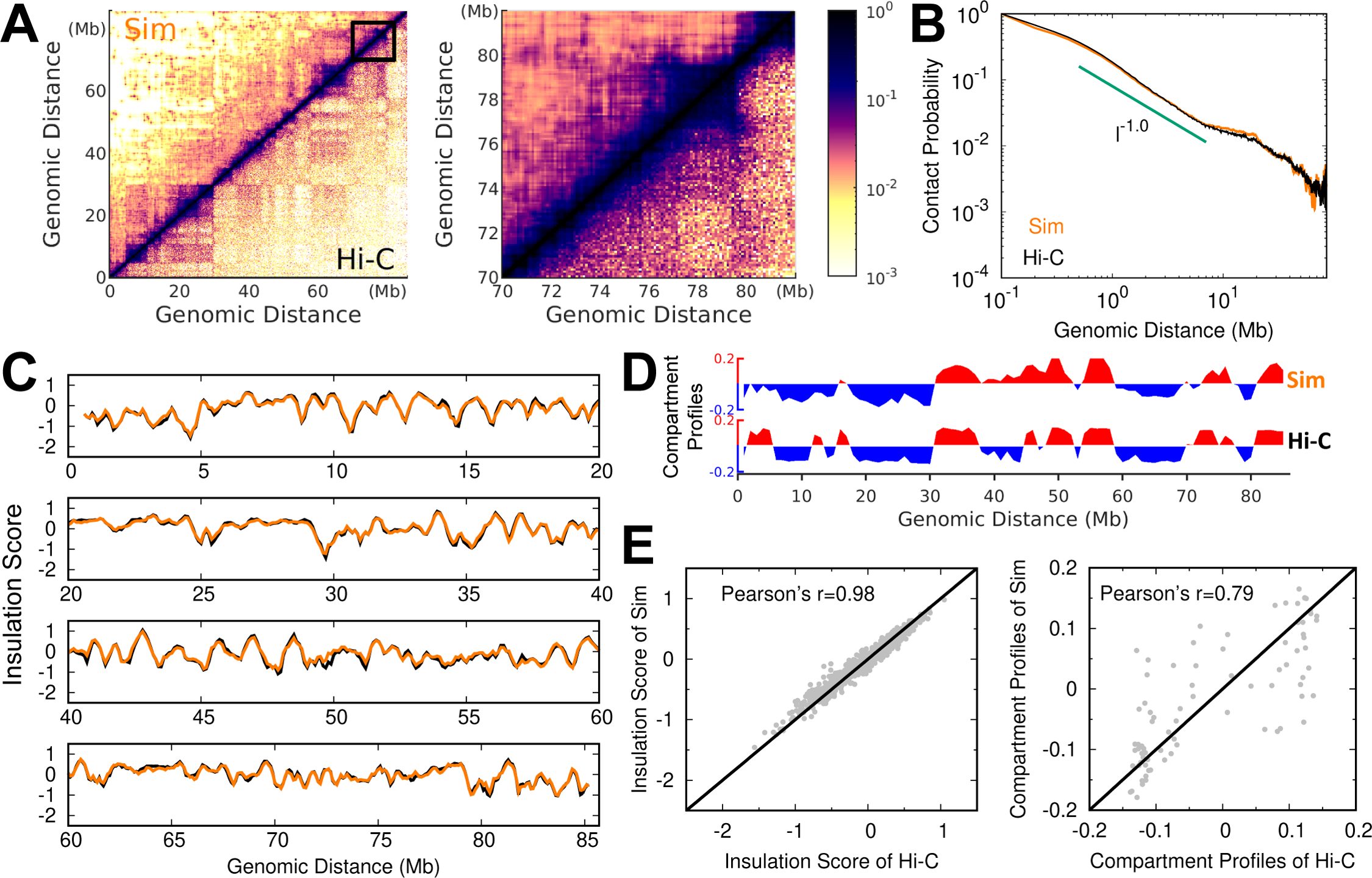

Supplement: S1 Fig — (A) Hi-C contact maps of the chromosome ensemble obtained from the simulations and the Hi-C data at the global (Left) and local (Right) scales. (B) Contact probability versus genomic distance in the chromosome for the simulations and the Hi-C data with a slope of -1.0 in the logarithmic scale at 0.5–7 Mb. (C) Insulation score of the chromosome obtained by the simulations and the Hi-C data. (D) Compartment profiles of the chromosome obtained by the simulations and the Hi-C data. (E) Correlations of insulation score (Left) and compartment profiles (Right) between the simulations and the Hi-C data. (TIF) [file pcbi.1009596.s002.tif]

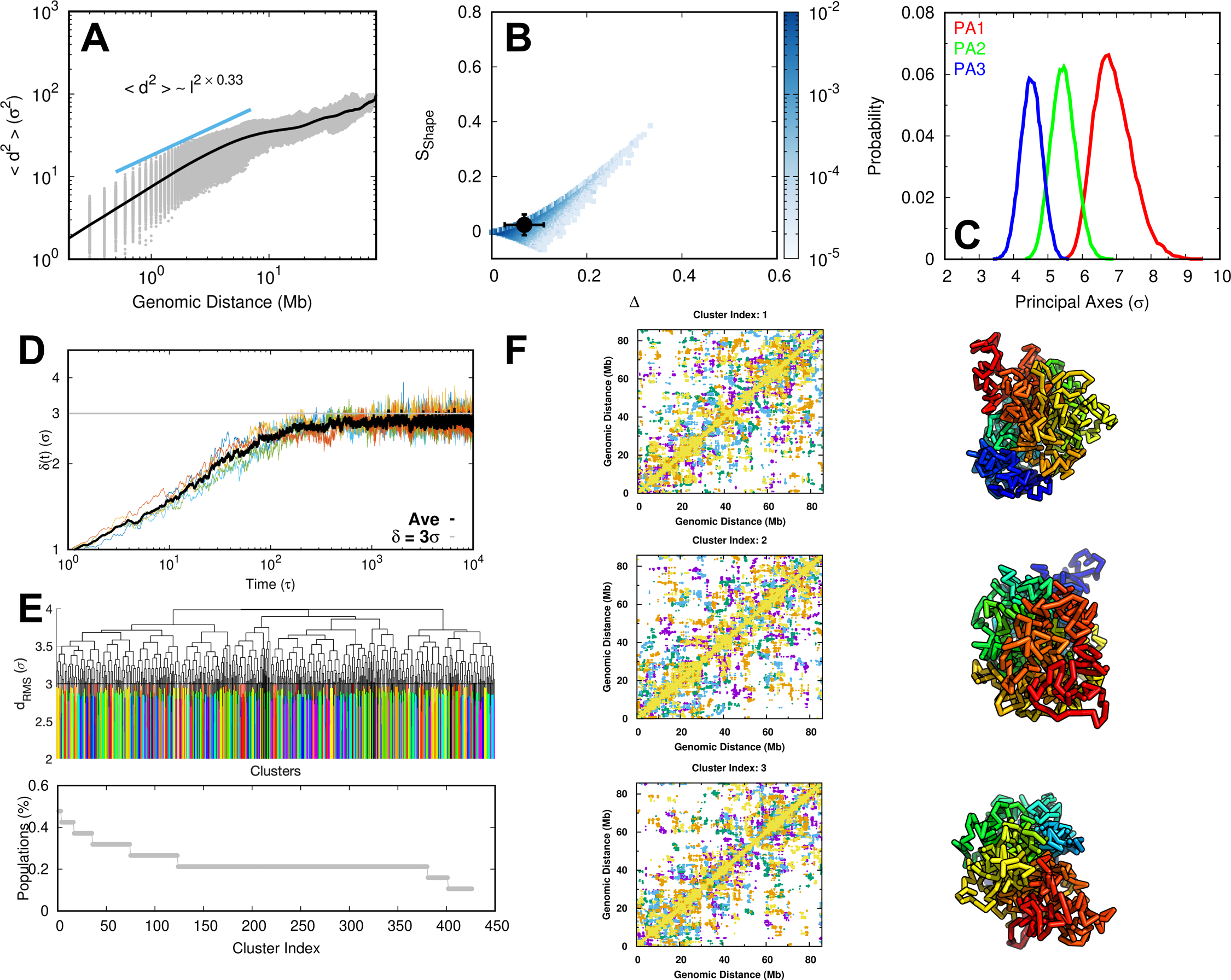

Supplement: S2 Fig — (A) Contact distance (d) versus genomic distance (l) in the chromosome. (B) The probability distributions of aspheric parameters of the chromosome. Δ and Sshape were calculated using the inertia tensor [84]. Deviation of Δ from 0 (the value corresponding to a sphere) gives an indication of the extent of anisotropy. Negative values of SShape correspond to oblate shapes and positive values of SShape to prolate shapes. (C) The probability distribution of the configurational extension on the three principal axes of the chromosome. (D) The time evolution of the average root mean square distance (drms) between every genomic pair in chromosome at the time t relative to its initial value: δ(t) = ∑i,j drms(i, j, t)/Nsum, where Nsum is the number of summed pairs. The maximum of δ(t) is close to and below 3σ. (E) The hierarchical clustering of the chromosome structural ensemble shown as a dendrogram (Top) and the populations of the cluster (Bottom). Cut-off distance 3σ was applied. (F) The top 3 most populated chromosome clusters. Each is shown with a mixed contact map (Left), which contains 5 structures within the cluster, and one representative structure (Right). (TIF) [file pcbi.1009596.s003.tif]

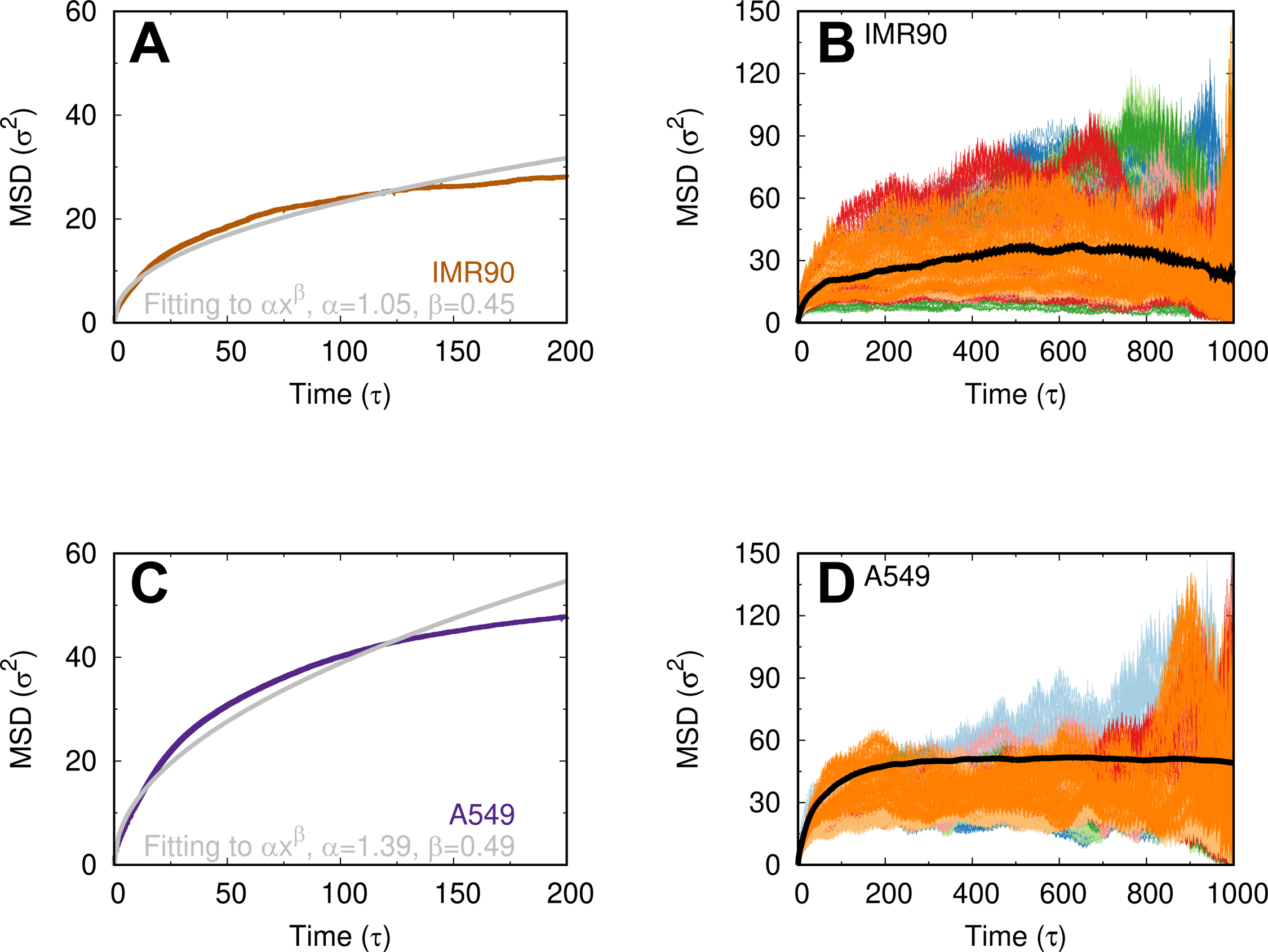

Supplement: S3 Fig — (A) Fitting of MSD to the power-law function (MSD ∼ αxβ) in the IMR90 cell. MSD was calculated by averaging the MSD from 5 independent simulations with the potential from the maximum entropy principle simulation V(r|IMR90). (B) MSD of all the individual chromosomal loci in the IMR90 cell state obtained from one simulation with average shown as the black line. (C) and (D) are same as (A) and (B) but for the A549 cell. (TIF) [file pcbi.1009596.s004.tif]

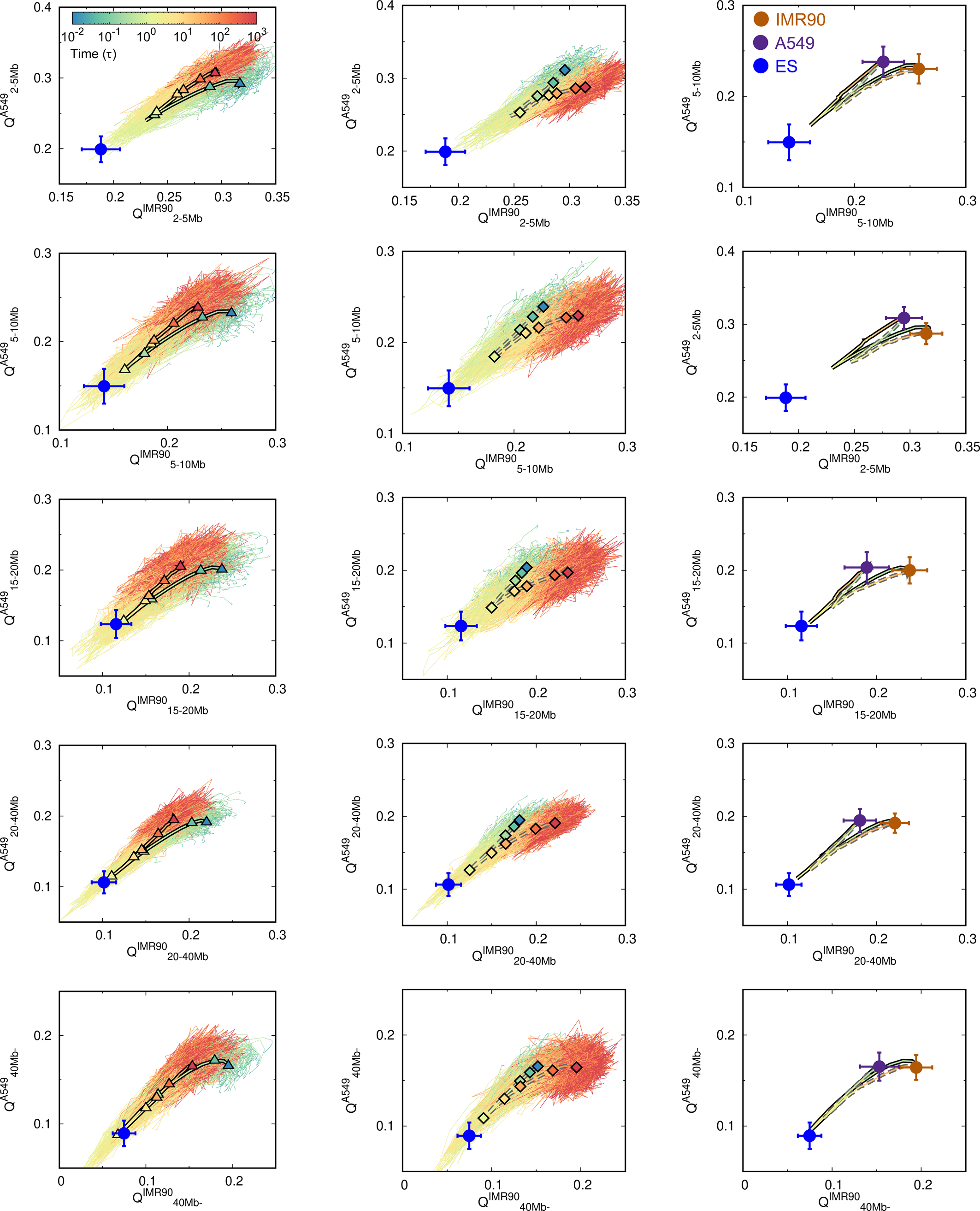

Supplement: S4 Fig — (TIF) [file pcbi.1009596.s005.tif]

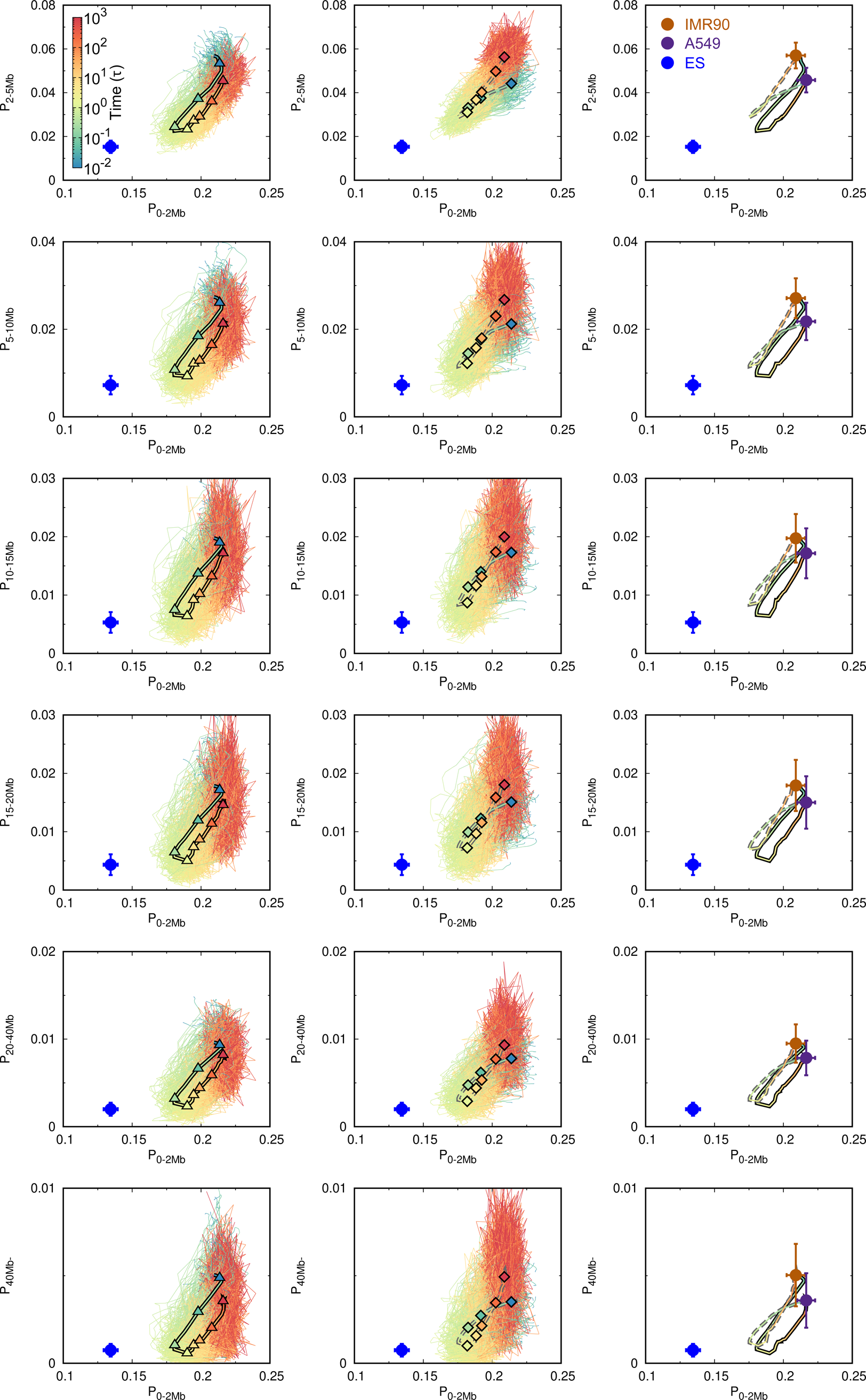

Supplement: S5 Fig — (TIF) [file pcbi.1009596.s006.tif]

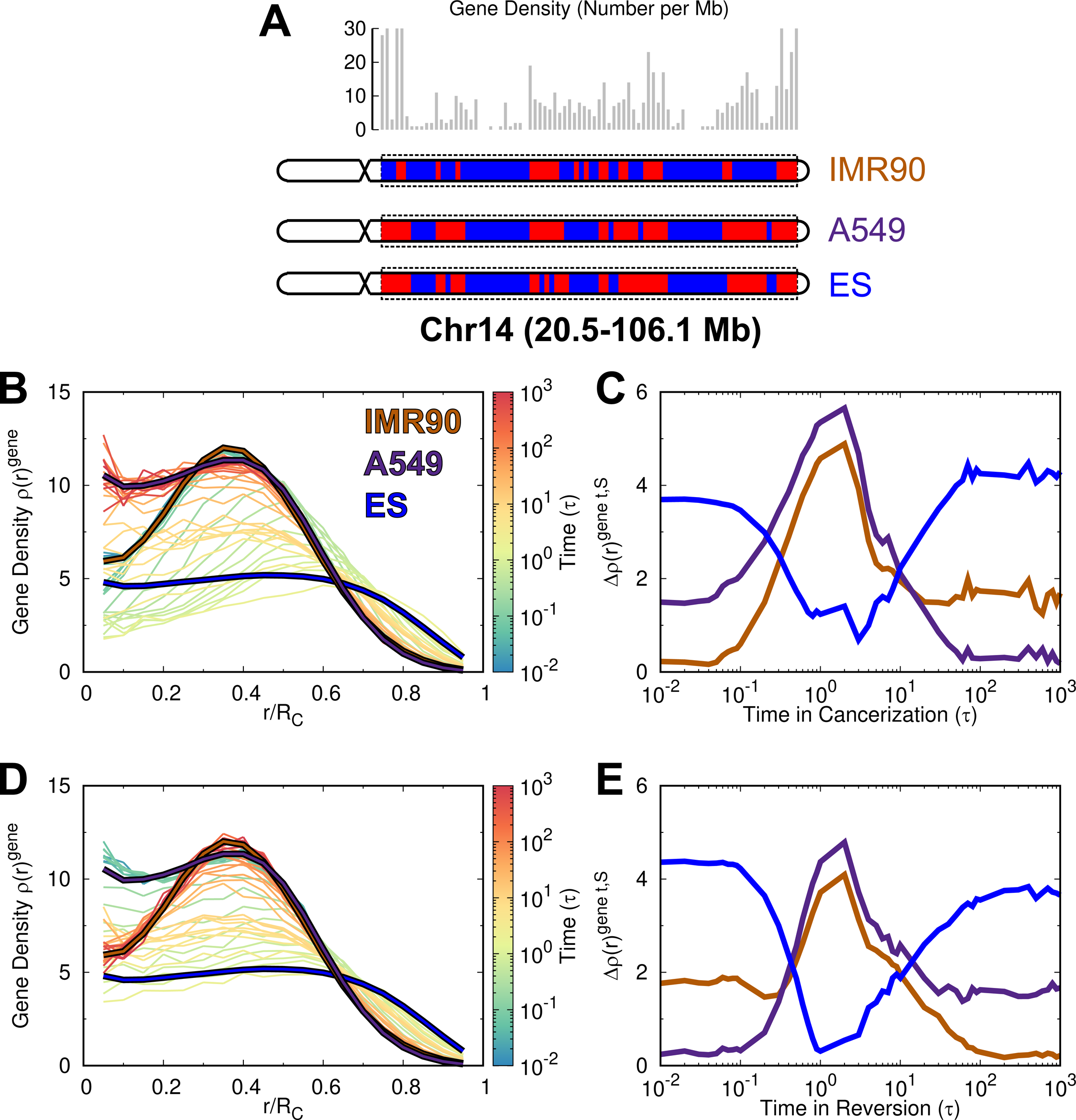

Supplement: S6 Fig — (A) Gene density along the chromosome and the compartment status of chromosomal loci at the IMR90, A549 and ES cells. Compartment A and B are colored red and blue, respectively. (B) The change of the radial gene density profile ρ(r)gene in the chromosome during cancerization. The profiles of the IMR90, A549 and ES cells are colored brown, purple and blue, respectively. (C) The difference of the radial density from the processing time to the reference cell (the IMR90, A549 or ES cell) during cancerization. (D) and (E) are the same with (B) and (C) but for the reversion process. The plotting details of (B-E) are the same with Fig 4, but for the radial density distributions of the genes. (TIF) [file pcbi.1009596.s007.tif]

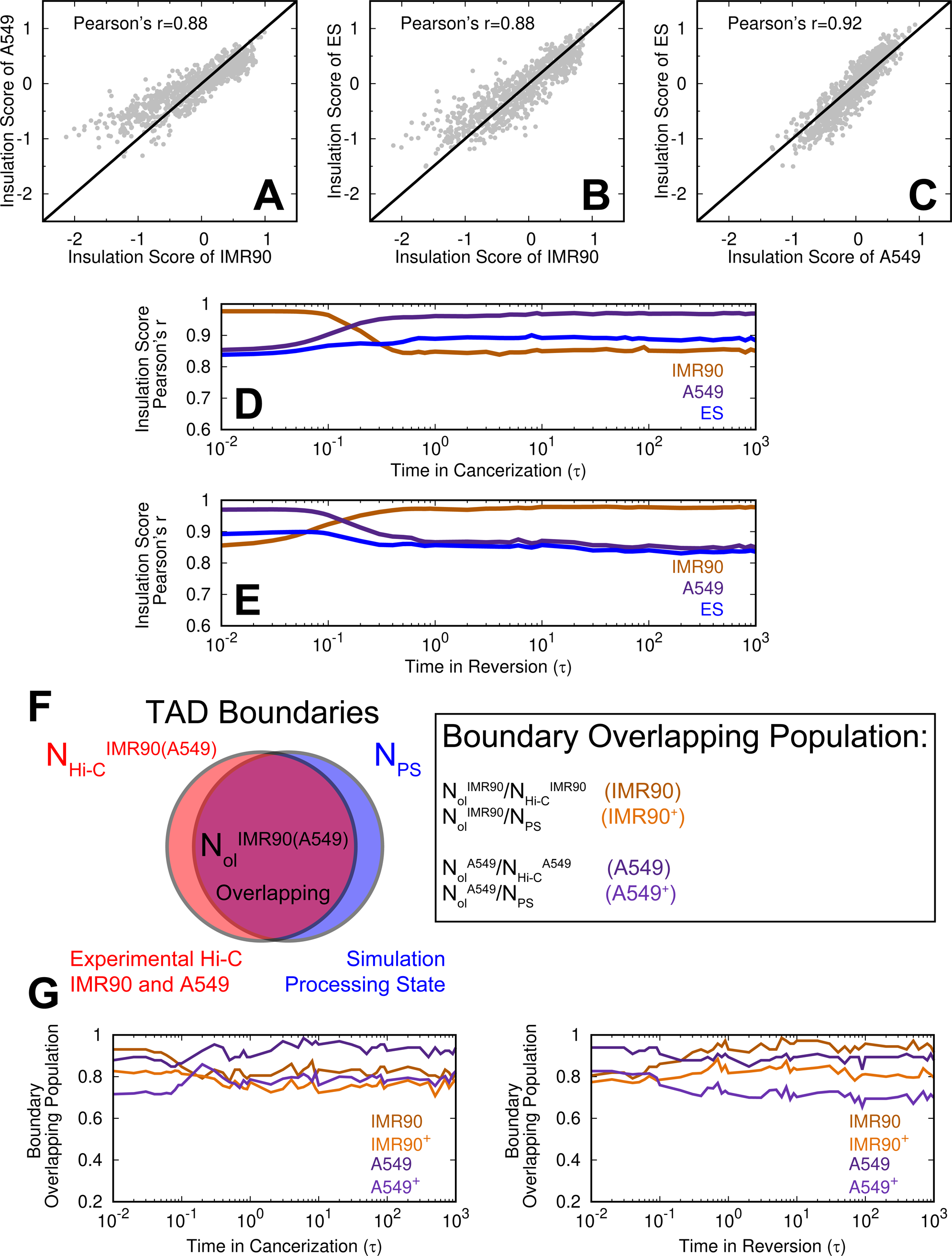

Supplement: S7 Fig — (A-C) The correlation between the insulation score among the IMR90, A549 and ES cells. The correlation coefficient of insulation score of the processing state during (D) cancerization and (E) reversion with those of the IMR90, A549 and ES cells. (F) An illustration of TAD boundary overlapping between the Hi-C data (IMR90 or A549) and simulation processing state during cancerization and reversion. The numbers of TAD boundaries detected by insulation score for experimental Hi-C (IMR90 or A549), simulation processing state, and the overlap between the Hi-C (IMR90 or A549) and processing state are denoted as NHi-CIMR90(A549), NPS, and NolIMR90(A549), respectively. (G) The change of TAD boundary overlapping population from the simulation processing state to the Hi-C of the IMR90 and A549 (calculated by NolIMR90/NHi-CIMR90 and NolA549/NHi-CA549) and from the Hi-C of the IMR90 and A549 to the simulation processing state (indicated by superscript “+” and calculated by NolIMR90/NPS and NolA549/NPS) during cancerization and reversion. (TIF) [file pcbi.1009596.s008.tif]

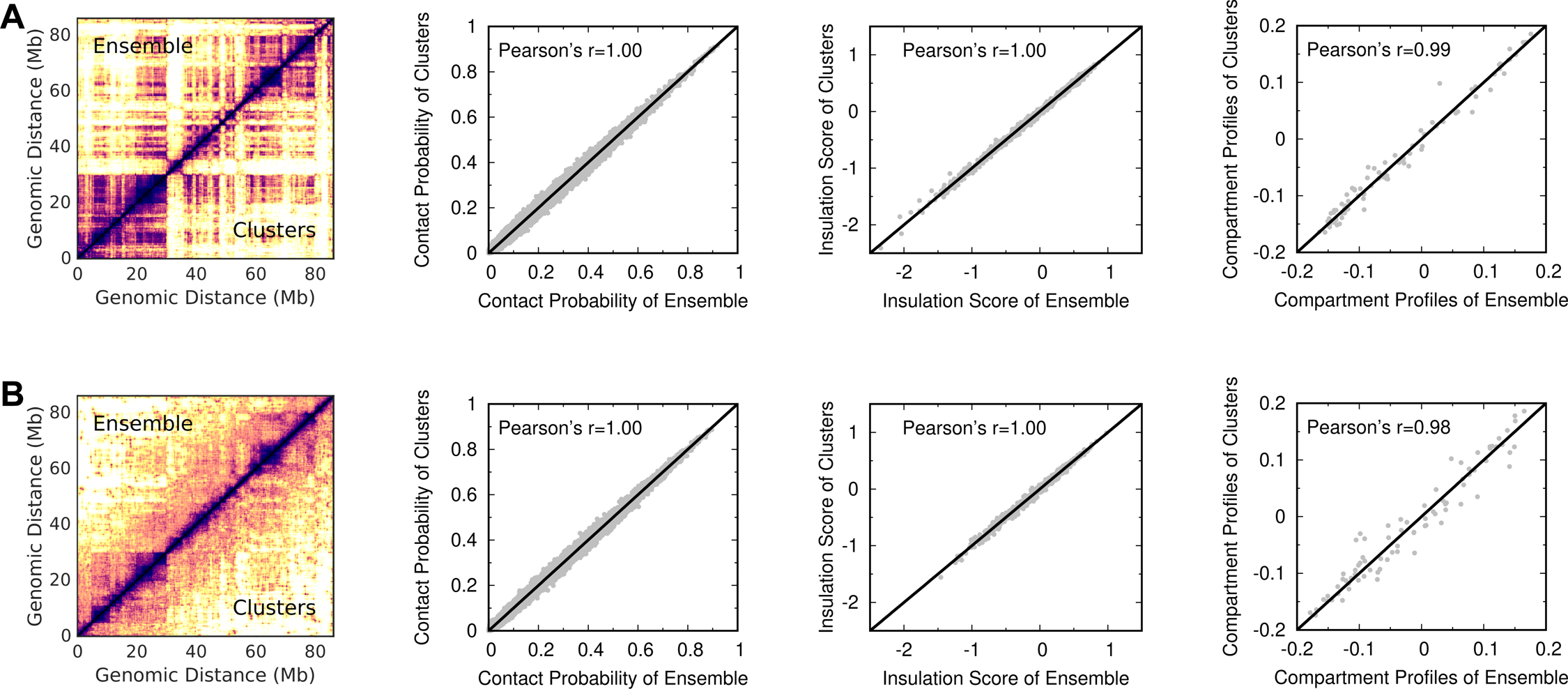

Supplement: S8 Fig — (A) The IMR90 cell. (B) The A549 cell. (TIF) [file pcbi.1009596.s009.tif]

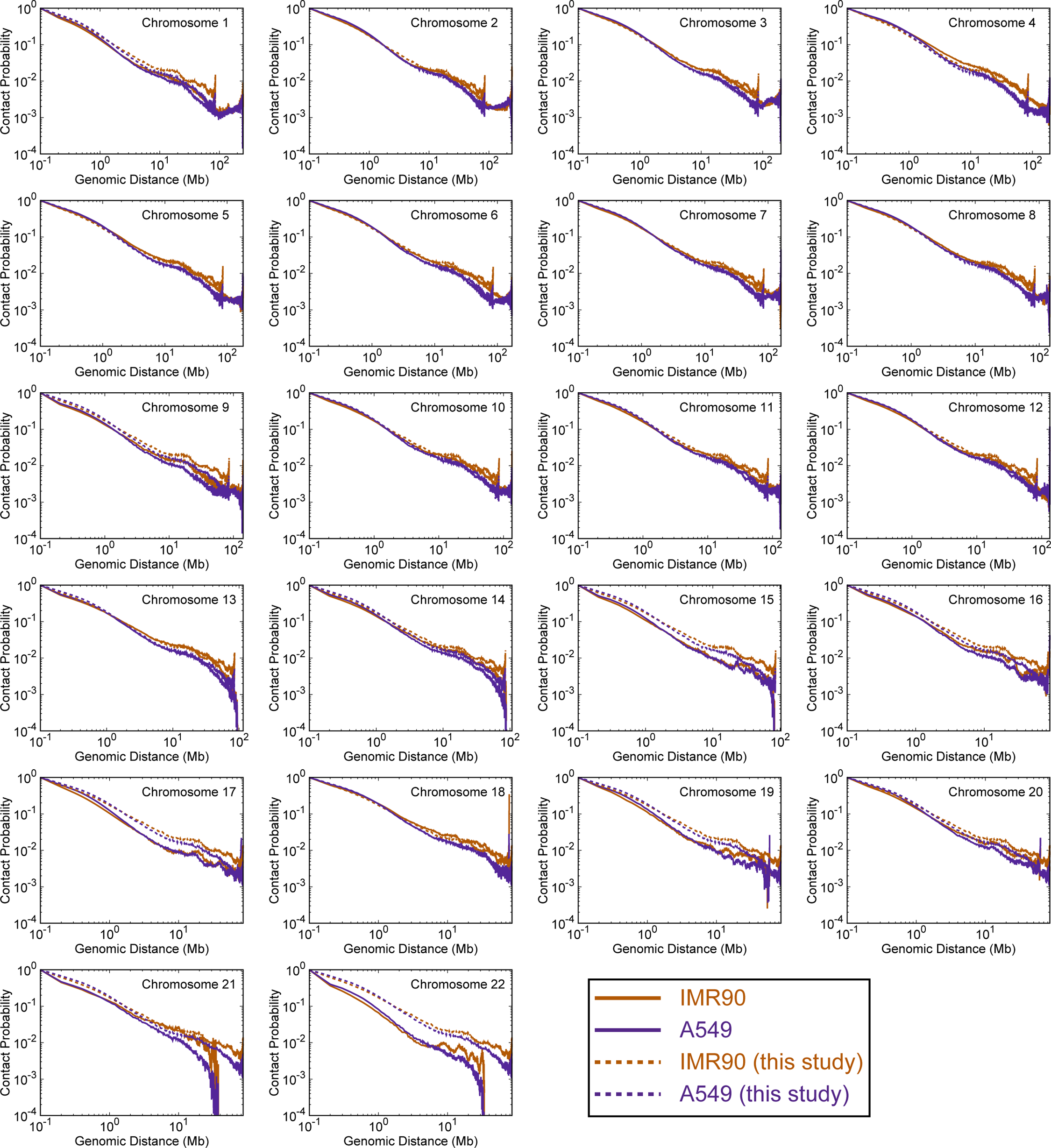

Supplement: S9 Fig — The dashed lines indicate the chromosome segment used in this study (chr14: 20.5-106.1 Mb). (TIF) [file pcbi.1009596.s010.tif]

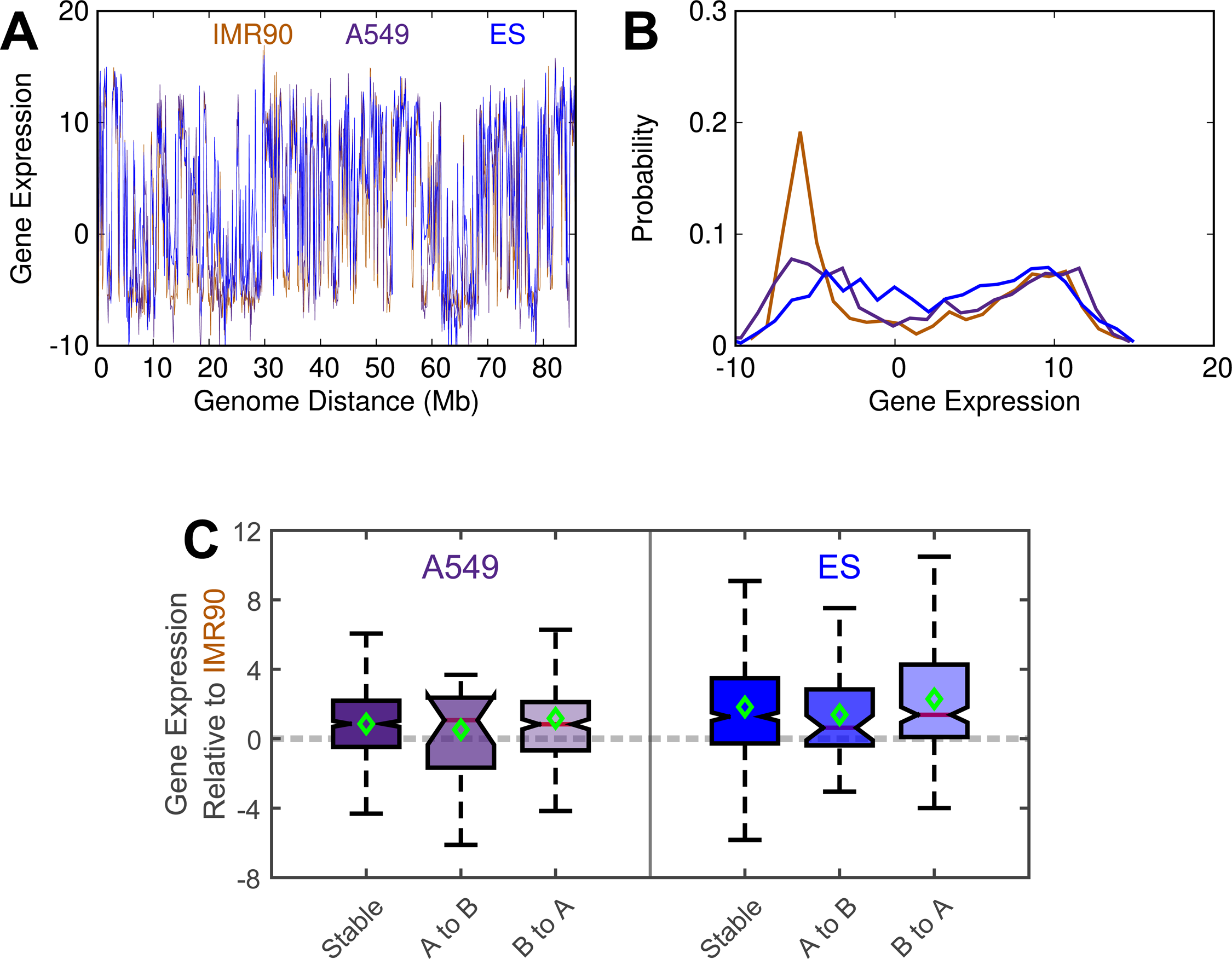

Supplement: S10 Fig — (A) Gene expression level along the chromosomal loci. Gene expression data were measured by the RNA-seq. The expression level for each bead in our model, which represents a DNA segment of 100 Kb in length, was determined as the sum of the reads in this 100 kb DNA segment. The value was further scaled by 106 to indicate the reads of “per million” and was represented in the logarithmic scale (log2). The calculation of the gene expression level is similar to the Reads Per Kilobase Million (RPKM), widely used in RNA-seq analysis. (B) Distributions of gene expression levels in the IMR90, A549 and ES cells. (C) Distributions of the changes in the gene expression levels for the genes that change the compartment status (“A to B” or “B to A”) or that remain the same (“stable”) when comparing the IMR90 cell to the A549 and ES cells, respectively. The red lines and green diamonds in the box plots indicate the median and mean values of the distributions, respectively. (TIF) [file pcbi.1009596.s011.tif]

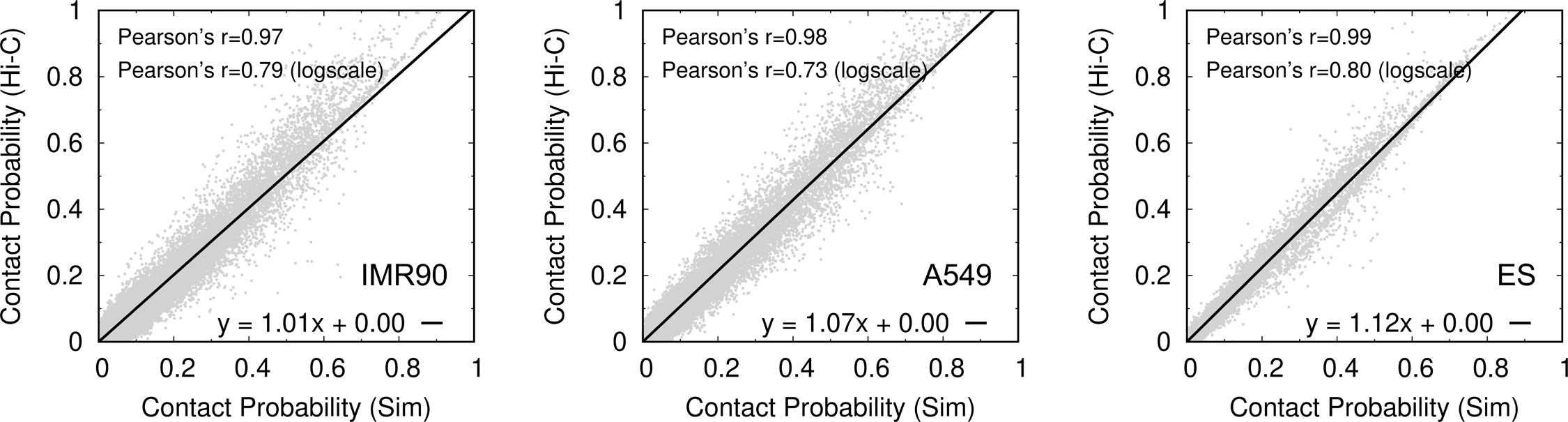

Supplement: S11 Fig — Pearson’s correlation coefficients were calculated based on the absolute values and logarithmic scaled values of contact probability. The calculations based on the logarithmic scaled values of contact probability were performed after removing the contacts with the probability equal to 0 in the Hi-C data. The linear fit of the simulated contact probability and the Hi-C data is shown. (TIF) [file pcbi.1009596.s012.tif]

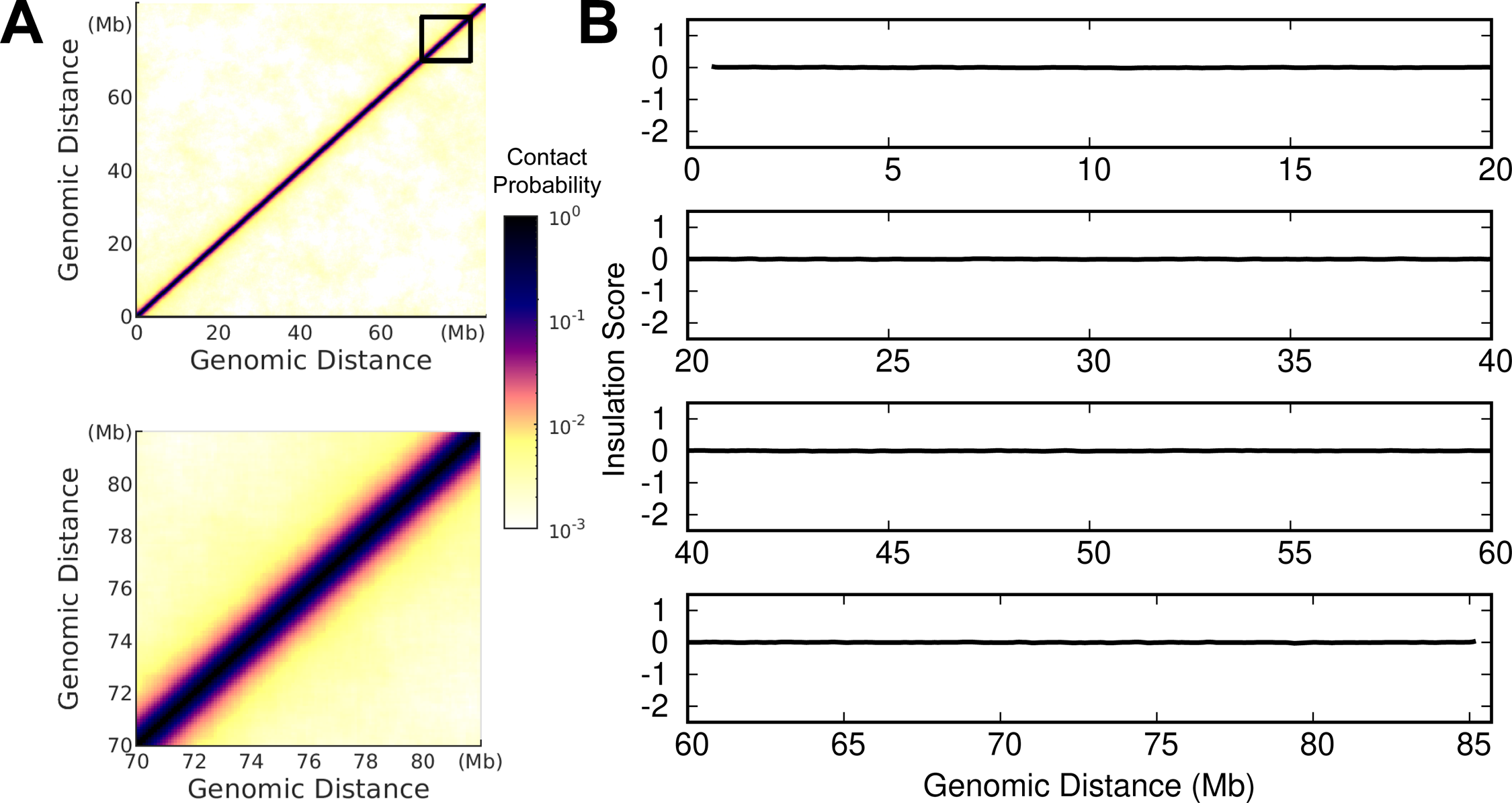

Supplement: S12 Fig — (A) The contact maps of the polymer at global (top) and local (bottom) scales. (B) Insulation score. (TIF) [file pcbi.1009596.s013.tif]
